# Bending-Twisting Motions and Main Interactions in Nucleoplasmin Nuclear Import

**DOI:** 10.1371/journal.pone.0157162

**Published:** 2016-06-03

**Authors:** Marcos Tadeu Geraldo, Agnes Alessandra Sekijima Takeda, Antônio Sérgio Kimus Braz, Ney Lemke

**Affiliations:** 1 Laboratório de Bioinformática e Biofísica Computacional, Departamento de Física e Biofísica, Instituto de Biociências de Botucatu, UNESP – Universidade Estadual Paulista, Botucatu, SP, 18618-970, Brazil; 2 Instituto de Biotecnologia (IBTEC), UNESP – Universidade Estadual Paulista, Botucatu, SP, 18607-440, Brazil; 3 Laboratório de Biologia Computacional e Bioinformática, Centro de Ciências Naturais e Humanas, UFABC – Universidade Federal do ABC, Santo André, SP, 09210-170, Brazil; University of Queensland, AUSTRALIA

## Abstract

Alpha solenoid proteins play a key role in regulating the classical nuclear import pathway, recognizing a target protein and transporting it into the nucleus. Importin-*α* (Imp*α*) is the solenoid responsible for cargo protein recognition, and it has been extensively studied by X-ray crystallography to understand the binding specificity. To comprehend the main motions of Imp*α* and to extend the information about the critical interactions during carrier-cargo recognition, we surveyed different conformational states based on molecular dynamics (MD) and normal mode (NM) analyses. Our model of study was a crystallographic structure of Imp*α* complexed with the classical nuclear localization sequence (cNLS) from nucleoplasmin (Npl), which was submitted to multiple 100 ns of MD simulations. Representative conformations were selected for calculating the 87 lowest frequencies NMs of vibration, and a displacement approach was applied along each NM. Based on geometric criteria, using the radius of curvature and inter-repeat angles as the reference metrics, the main motions of Imp*α* were described. Moreover, we determined the salt bridges, hydrogen bonds and hydrophobic interactions in the Imp*α*-NplNLS interface. Our results show the bending and twisting motions participating in the recognition of nuclear proteins, allowing the accommodation and adjustment of a classical bipartite NLS sequence. The essential contacts for the nuclear import were also described and were mostly in agreement with previous studies, suggesting that the residues in the cNLS linker region establish important contacts with Imp*α* adjusting the cNLS backbone. The MD simulations combined with NM analysis can be applied to the Imp*α*-NLS system to help understand interactions between Imp*α* and cNLSs and the analysis of non-classic NLSs.

## Introduction

Solenoid proteins are molecules composed of structural motifs that are arranged in tandem, creating a superhelical architecture. This modular characteristic provides the establishment of folding and binding contacts that contrasts the globular proteins, allowing higher flexibility and the arrangement of cooperative protein-protein interactions due to the formation of diversified interfaces [1–4].

A remarkable characteristic in nuclear protein import regulation is the contribution of solenoid proteins to recognizing a target protein and transporting it into the nucleus [5]. One of the most studied pathways is the classical nuclear import pathway, which requires the interaction between the solenoid Importin-*α* (Imp*α*) and Importin-*β* (Imp*β*) proteins, followed by the assembly of the cargo protein to Imp*α* [6–10]. This trimeric complex is translocated through the nuclear pore complex (NPC), and the cargo protein is delivered into the cell nucleus mediated by Ran-GTP-dependent steps of protein-protein interactions [11, 12]. The dissociation of the cargo protein from Imp*α* is catalyzed by nucleoporins (e.g., NUP50) [13], and Imp*α* binds to its export factor CAS complexed with RanGTP [14]. A final step is the recycling of both Imp*α* and Imp*β* back to the cytoplasm.

Imp*α* is composed of ten tandem repeats of armadillo motifs (ARM) oriented in an elongated and curved-twisted shape [6, 10, 15]. Each motif is formed by a superhelical architecture of three *α*-helices [6, 15, 16]. From the curved orientation, convex and concave surfaces can be identified; in particular, the inner concave surface harbors conserved residues that mediate the cargo protein binding [6, 17].

Cargo protein transport depends on the recognition of a specific sequence signal by Imp*α* called the nuclear localization sequence (NLS). For classical NLSs (cNLSs), the binding pattern to Imp*α* is primarily mediated by one or two clusters of positively charged residues, the monopartite or bipartite cNLSs, respectively [18–22]. The clusters of bipartite cNLSs are separated by 10–12 variant residues, denominated as the linker region.

The inner concave surface of Imp*α* is adapted to receive either monopartite or bipartite cNLSs, and its specific binding sites can be identified as the major and minor sites. Structural and biophysical studies have related important positions of Imp*α* to the NLS binding: positions P2–P5 from the major site (ARMs 2–4; binding to both monopartite and bipartite cNLSs) and P1’-P2’ from the minor site (ARMs 6–8; binding preferentially to bipartite cNLSs) [21, 23]. Moreover, the region between major and minor sites (ARMs 4–6) provides interactions and is also considered fundamental in cNLS recognition [21, 24–30].

Few studies have reported the flexibility and structural integrity of Imp*β* [2, 31]; however, the application of computational simulation approaches is underexplored for understanding the wide structural motions and the interaction basis of nuclear import mediated by Imp*α* binding. Therefore, two main questions arose: (i) Are there motions related to NLS recognition? (ii) What is the dynamical behavior of the interactions on the complex interface? Motivated by these questions, we combined molecular dynamics simulations (MD) and normal mode (NM) analysis. As our cNLS model of study, we used the crystallographic structure of nucleoplasmin NLS (NplNLS) complexed with Imp*α* because experimental works involving Npl [17, 24, 32] are available and could be used to support and contrast with our simulation results. Npl is the first protein to be described as a molecular chaperone involved in chromatin reprogramming [33], and it is characterized as containing a bipartite cNLS (Npl:^151^GSAV**KR**PAATKKAGQA**KKKK**LD^172^; residues in bold indicate the positions in contact with Imp*α* minor and major binding sites, respectively). We determined that bending and twisting-like major movements of Imp*α* may influence the NLS binding. In addition, we confirmed the importance of contacts in the major and minor sites, along with contacts flanking these sites, including the linker region, for the establishment of carrier-cargo recognition.

## Materials and Methods

### Model of study: Classical bipartite NLS

All simulation analyses were conducted using the crystallographic structure of NplNLS complexed to mouse Imp*α* isoform 2 (Imp*α* -NplNLS; PDB ID: 3UL1) [17]. The missing atoms from residues G^152^ and S^342^ of NplNLS (^152^GSAVKRPAATKKAGQAKKKKLD^172^) and Imp*α* (Imp*α*ΔIBB, residues 72–497), respectively, were modeled with MODELLER v9.11 software [34]. The N-terminal Imp*β*-binding (IBB, residues 1 to 71) domain from Imp*α* was kept truncated because it competes in the binding in the NLS region. The choice criteria for determining the best model were guided by the correct stereochemistry and the occurring interactions in the complex interface, using Molprobity [35] and PISA [36] servers, respectively. An additional modeling for Imp*α* was conducted removing the peptide (Apo Imp*α*) to compare the motions and flexibility of Imp*α* in the presence and absence of the cNLS.

### MD simulations

The topology and parameter files were generated on the program GROMACS v4.5.3 [37], employing the force field Charmm36 [38] without a protonation requirement because the PROKPA webserver (http://propka.org) analysis indicated no protonation changes. A cubic box of 56,568 explicit TIP3P water molecules [39, 40] was generated, ensuring at least 10 ångströms (Å) from the protein system to each edge of the box. Counter ions were added for system-charge neutralization by replacing water molecules. The system was submitted to a gradual energy minimization composed of four steps: (i) 500 steps of energy minimization by the steepest descent method, limiting the protein and peptide movement to accommodate the solvent molecules; (ii) 50,000 steps of energy minimization by the steepest descent method, limiting the movement of the protein and peptide’s main chains; (iii) 50,000 steps of unrestricted energy minimization by the steepest descent method; and (iv) 50,000 steps of unrestricted energy minimization by the conjugated gradients (CG) method.

Equilibration and unrestrained MDs were performed in periodic boundary conditions. The leapfrog integrator was used for integrating Newton’s equations of motion. The linear constraint solver (LINCS) method [37, 41] was used to freeze bonds involving hydrogen atoms, allowing an integration step of 2 femtoseconds (fs). The cutoff distance for short-range electrostatic and van der Waals interactions was 10 Å. The Particle Mesh Ewald method (PME) [42] was used to treat long-range electrostatics.

The system was equilibrated in two steps, both applying a position restraining force on the heavy atoms of the protein. The first stage involved the adoption of NVT conditions (constant number of particles, volume and temperature), heating the system to the target temperature of 300 kelvin (K) and simulating in this condition by 100 picoseconds (ps) with a velocity-rescaling (V-rescale) thermostat [43]. The second stage involved 100 ps of equilibration by the adoption of NPT conditions (constant number of particles, pressure and temperature), with pressure coupling using the Parrinello-Rahman barostat [44] and keeping the pressure relatively constant, close to the value of 1 bar. After the equilibration procedures, the restraints were removed, and the system was submitted to three MD simulations of 100 nanoseconds (ns) each, with structure sampling every 10 ps.

To ensure the conformational sampling of the system, a clustering technique proposed by Lyman and Zuckerman [45] was applied. In this approach, the steps below were applied to generate a collection of reference structures of the simulations {*S*_*i*_}: (1) A cutoff root-mean square deviation (RMSD) *d* was defined. (2) Each simulation was merged into a single trajectory file. (3) One structure from the trajectory file was sampled randomly and denominated as the reference structure. (4) All structures were compared to the sampled reference structure, and the ones with an RMSD less than *d* were removed from the trajectory file. (5) Steps 1, 2, 3 and 4 were repeated until all structures were removed, thus generating a collection of reference structures {*S*_*i*_}. (6) Based on the collection {*S*_*i*_}, each frame from the trajectory file was clustered with the nearest reference structure, and the frequency of structures of each cluster was then calculated. An estimation of convergence is assessed when each reference structure is equivalently represented in each simulation. The parameter for the calculation of *d* was the global RMSD of carbon-*α* (C*α*) atoms. The choice of *d* (= 2.5 Å) was constrained to a feasible number of reference structures for the subsequent analysis of NMs.

### NM analysis

Each reference structure obtained from MD simulations was minimized in the GROMACS program with explicit solvent using the same methodology described above. All NM analyses were carried out in the CHARMM v.36b1 program [46]. The topology and parameter files required for CHARMM were generated with the CHARMM-GUI server (www.charmm-gui.org) employing an additional energy minimization. The CG algorithm was applied with harmonic constraints that were progressively decreased from 250 to 5 kcal/mol^-1^Å^-2^, with 100 steps of minimization at each decrease. Then, the constraints were removed, and 10,000 steps of CG were carried out. Afterwards, the adopted basis Newton Raphson (ABNR) algorithm was applied with no constraints for 300,000 steps.

The final minimized structure was used for the calculation of the 87 lowest frequency NMs, using the VIBRAN module of CHARMM for each reference structure. An NM-displacement method using the VMOD facility of CHARMM was applied, generating structures along each NM based on short MD simulations at a low temperature (30 K), followed by energy minimization. Based on the values of mass-weighted root mean square (MRMS), the maximum displacement range was set to 3 Å for each direction of the NM with a 0.1 Å projection step, totaling 61 structures per NM. For each MRMS step, a harmonic force constant over the C*α* atoms was applied (increasing from 1,000 until 10,000 kcal/mol^-1^Å^-2^), and a short MD simulation was carried out for 1 ps for each constant value, totaling 10 ps of simulation. Keeping the restraints, 1,000 steps of CG energy minimization were employed to generate the final structure. In addition, for each projection step, a value of the total restraint energy, according to the miscellaneous mean field potential (MMFP) facility of CHARMM, was used as a criterion for discarding unfavorable conformations.

### Data Analysis

#### PCA over MD simulations

Principal component analysis (PCA) from MD simulations was performed using the quasi-routine of the module VIBRAN of CHARMM to obtain the covariance matrix of C*α* atomic displacements from the trajectories and identify the most relevant structural variations. The calculation was performed according to the description of Floquet et al. [47], and the resulting principal components (PCs) were used to compare the motions observed in NMs.

#### Collectivity

The measurement of the involvement of atoms in a particular protein motion (referred to as degree of collectivity) for a given NM was calculated according to Bruschweiler [48] and Tama and Sanejouand [49], using an *in-house* CHARMM script. The degree of collectivity is comprised between 0 and 1. Values close to 1 indicate maximum collectivity.

#### Geometric analysis of Imp*α*

**Fig 1:**
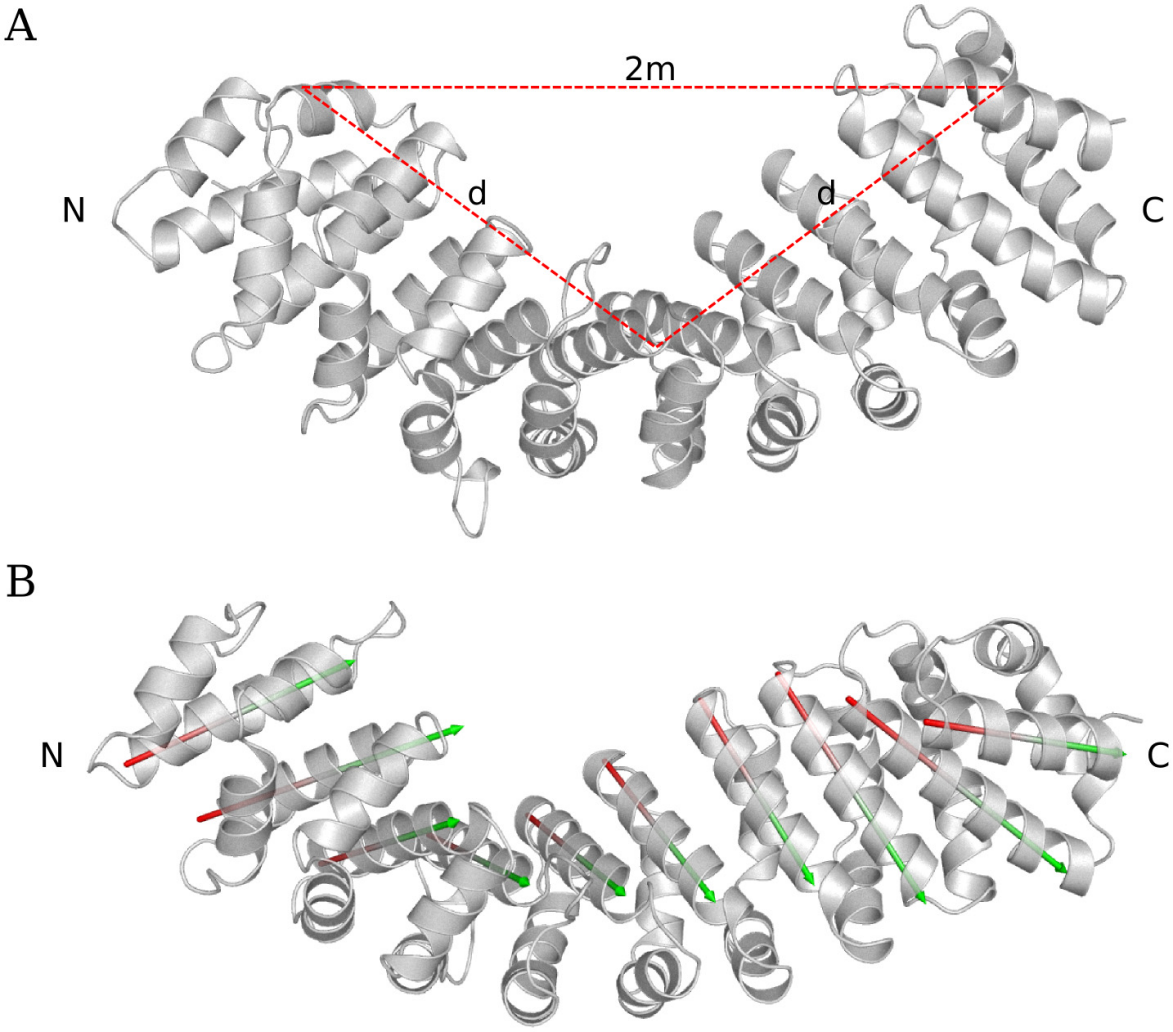
Scheme of geometrical methods adopted for the description of the Imp*α* motions. (A) Display of the selected vertices and the determined distances (2m and d) for the calculation of the radius of curvature. (B) The vectors generated to H3 for each ARM repeat.

The wide motions from Imp*α* were described in terms of geometrical measurements. For bending characterized motions, three vertices (represented by Imp*α* residues R^117^, A^313^ and K^486^) were manually selected: two vertices in distal tips and one vertex at the middle point of the protein. The plane formed by these three vertices corresponded to the plane of the observed bending motion (Fig 1). Then, the radius of curvature (*R*) was calculated:

$$R=\frac{d^{2}}{2\sqrt{d^{2}-m^{2}}}$$ (1)

*d* is the distance from the distal vertices to the middle vertex, and *m* is half the distance between the two distal vertices.

For twisting characterized motions, the angle between *α*-helices 3 (H3) from neighboring ARM repeats were calculated using an available script for PyMol (http://www.pymolwiki.org/index.php/AngleBetweenHelices). For each pair of helices, vectors were defined along the C*α* atoms, and the torsion angles between these vectors were determined.

This geometric analysis was also applied to X-ray-solved structures of Imp*α* complexed with different types of bipartite NLSs. These structures were retrieved by the basic local alignment search tool allocated at Prody software [50] using the Imp*α* -NplNLS model as the query. Only structures with 100% of sequence identity to Imp*α* were selected to be compared to the simulation data.

#### Maps of cross-correlations

The initial comparison of residue-residue contacts from the MD and NM results was initially performed by the generation of maps of cross-correlation to evaluate the associated movements between NplNLS and Imp*α*. The calculation of correlations corresponded to the ensemble of the trajectories of all MD simulations into one final pseudo-trajectory. Similarly, an ensemble of all structures from NM-displacements into one pseudo-trajectory was conducted. The cross-correlation calculations were performed in the Wordom software [51].

#### Interactions evaluation

The occurrence of specific interactions in the Imp*α*-NplNLS interface was evaluated. The determination of salt bridges and hydrogen bonds were performed using the VMD software [52]. The criteria for considering the occurrence of these interactions were the donor-acceptor distance for salt bridges and hydrogen bonds ⩽3.5 Å and the donor-hydrogen-acceptor angle deviation for hydrogen bonds ⩽60 degrees. The determination of hydrophobic contacts was based initially on the LIGPLOT program, using the crystallographic and *in silico* model of Imp*α* -NplNLS to generate a list of possible interactions. Later, based on this list, the distances of the closest carbon atom from the hydrophobic side chains of each residue-pair were calculated for the MD and NM ensemble pseudo-trajectories to calculate the percentage of the occurrence of each hydrophobic contact. The criterion adopted was distances ⩽4 Å.

#### Complementary analysis

The backbone RMSD and the C*α* root-mean-square fluctuations (RMSF) of MD and NM-displacements were calculated in the Wordom software [51]. The structural alignment of NplNLS peptides was performed in the Crystallographic Object-Oriented Toolkit (Coot) software [53]. All graphics from the calculations above were performed using the R software [54], and the structural analysis, visualization and generation were performed in the Pymol software [55].

## Results

### Selection of Imp*α* -NplNLS model

A stereochemical analysis allowed for the selection of the best complex model, taking into account the geometry and maintenance of the main interactions in the peptide-protein interface. The best-selected model showed a Ramachandran plot for Imp*α* with 96.7% and 3.3% of the residues in favored and allowed regions, respectively, without residues in the outlier region, whereas NplNLS had 100% of the residues in favored regions.

**Fig 2:**
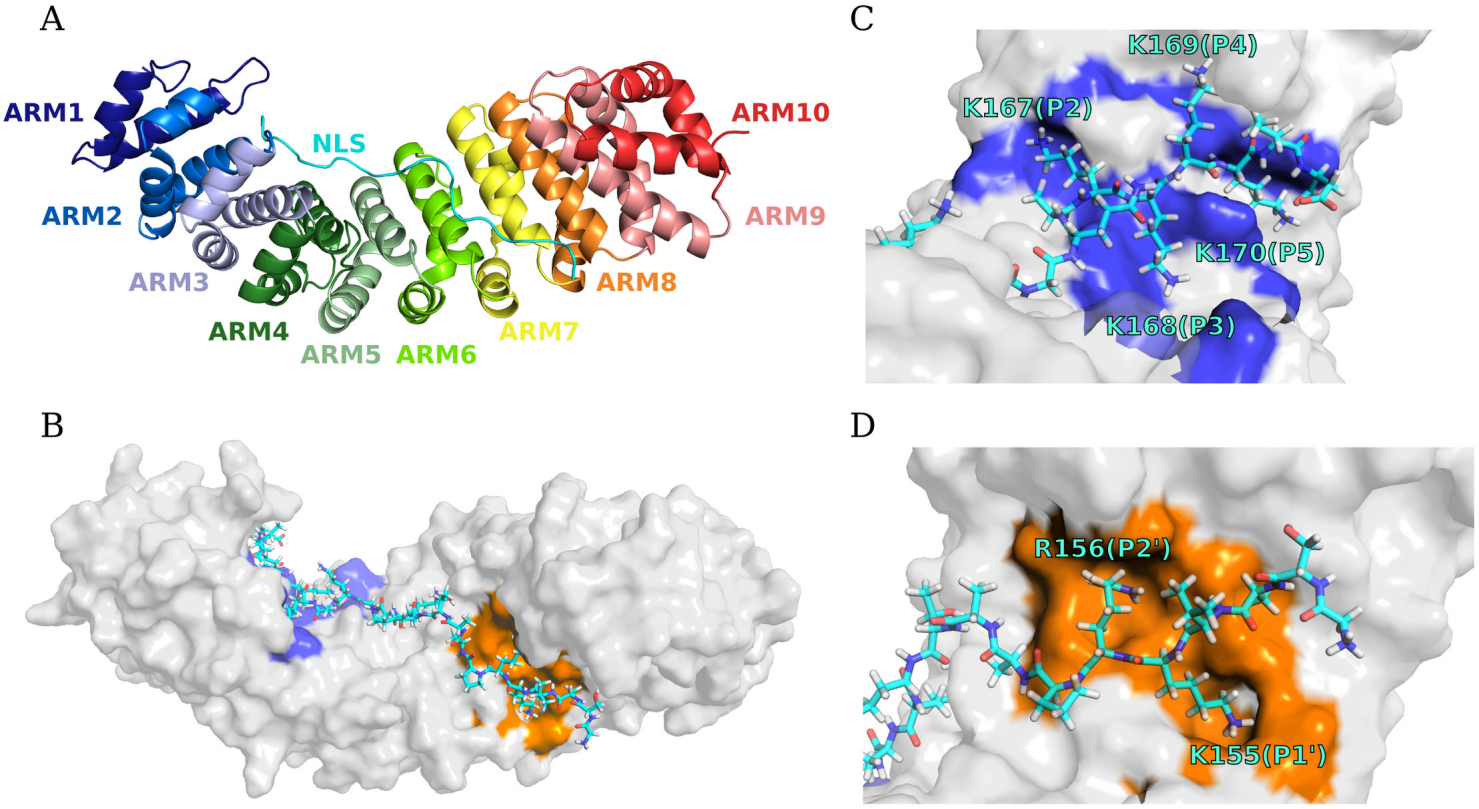
The starting structure of Imp*α*-NplNLS for MD simulations. (A) The Imp*α* as a cartoon diagram colored based on each ARM repeat as a rainbow spectrum from N-terminal (blue) to C-terminal (red) and the NplNLS as a cyan cartoon diagram positioned in an antiparallel configuration compared to Imp*α*. (B) The surface representation of Imp*α* with the NplNLS as a cyan stick diagram, indicating both major (blue) and minor (orange) binding sites. (C) The major site zoom indicating positions P2–P5 and (D) the minor site zoom in P1’ and P2’. In both sites, the positively charged side chains are positioned in the main pockets of the Imp*α* binding core.

An overall evaluation of the Imp*α* -NplNLS model indicated the NplNLS harboring in both major and minor binding sites of Imp*α*, with the main residues inside the binding pockets (Fig 2). Furthermore, we were able to evidence the previously stated interactions in the interface of this complex [17, 24]; the only major exception was the absence of _Imp*α*_D^325^ in contact with _Npl_K^155^ in the minor site (S1 Fig).

### Standard MD combined with NM-displacement method

The trajectories obtained from the three Imp*α*-NplNLS MD simulations were clustered into three reference structures (67,730 ps, 207,080 ps and 274,970 ps), each one exhibiting a similar frame-frequency among simulations, indicating a likely convergence of the MDs (S2 Fig). Reference structure 207,080ps was the most representative in the simulations (approximately 80% of the trajectories clustered with this structure) and was more similar to the X-ray-solved NplNLS structure based on the backbone RMSD values (S1 Table) obtained from the structural alignment of the NLSs (S3 Fig). In this alignment, important positions from major and minor sites were occupied by the expected residues, with similar side chain conformations, represented by _Npl_K^167^, _Npl_K^168^ and _Npl_K^170^ in positions P2, P3 and P5, respectively, and _Npl_K^155^ and _Npl_R^156^ in P1’ and P2’, respectively. Reference structures 67,730 ps and 274,970 ps showed a greater structural variance of side chains in the major site than in the minor site.

The subsequent step was the NM-displacement method applied to the three MD reference structures. We observed flexible and favorable motions in the early NMs (modes 7–20) of Imp*α*-NplNLS, represented by lower values of restriction energy along the whole displacement range, compared to the remaining modes (blue areas in the S4 Fig). Similar patterns were also observed for Apo Imp*α*; however, the favorable motions were extended up to NM29 (S5 Fig), indicating that more conformations could be acquired favorably compared to the Imp*α* bound to NplNLS.

The distribution of backbone RMSD showed that standard MD in combination with the NM-displacement method increased the conformational exploration, reaching values over 5 Å, particularly to reference structures 207,080 ps and 274,970 ps, whereas MD alone reached only approximately 2.5 Å (S6 Fig). Moreover, greater C*α* fluctuations were observed in the NM-displacement technique, based on the RMSF values (S7 and S8 Figs). The comparison between the RMSF values for Imp*α*-NplNLS and Apo Imp*α* showed small differences, primarily limited to the region of the major site (within the range of residue 100 to 200), where Apo Imp*α* was more flexible (S9 Fig).

### Collective motions of Imp*α*

**Fig 3:**
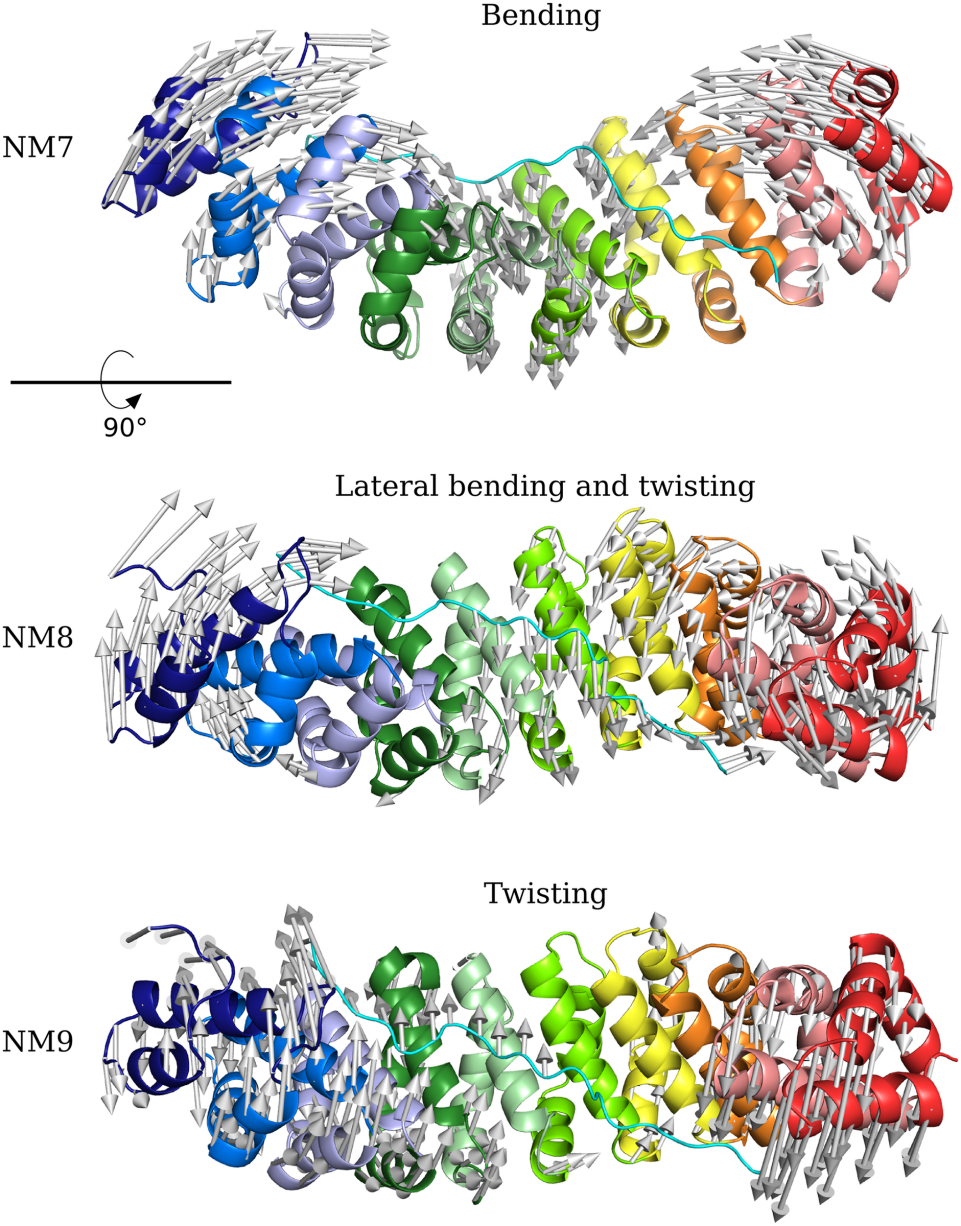
Main motions observed from NM analysis of Imp*α* -NplNLS. Imp*α* (cartoon model) is shown as a rainbow spectrum from N-terminal (blue) to C-terminal (red), and NplNLS (cyan cartoon model) is positioned in an antiparallel configuration compared to Imp*α*. The vector arrows for NM7–9 are shown with the correspondent description of the motion that they described. NM7 is shown in a front view, whereas NM8 and NM9 are shown in an upper view (90° rotation in the X-axis).

The description of the motions obtained from MD and NM calculations combined the results from a qualitative vector-based analysis and a quantitative geometrical analysis. The first NMs showed wide and collective types of motions of the ARM repeats of Imp*α*, primarily along modes 7–17 (S10 Fig). A qualitative analysis of vectors from modes 7 and 9 clearly showed a motion pattern, described by a bend and a twist (Fig 3 and S1 and S3 Movies), respectively.

**Fig 4:**
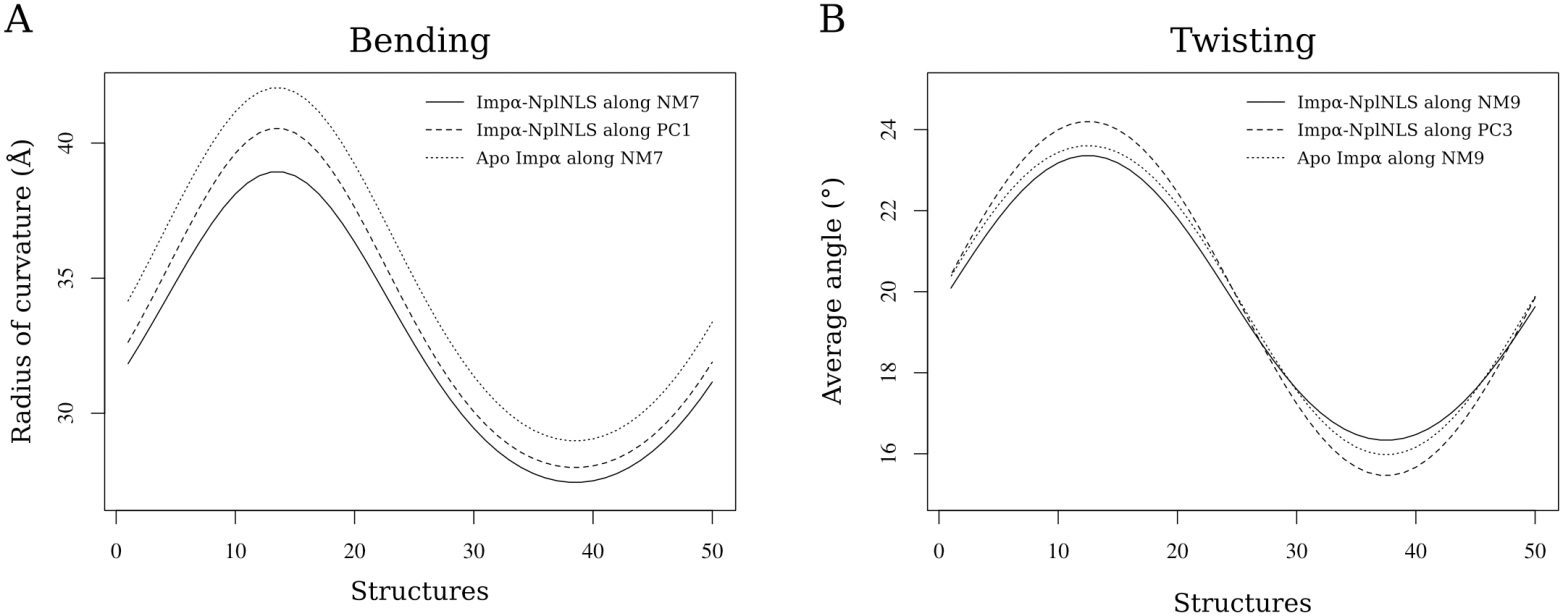
Geometric analysis of the bending and twisting motions of Imp*α*. (A) The bending motion was quantitatively characterized by the radius of curvature along NM7 (solid line) and PC1 (dashed line) for Imp*α* -NplNLS and along NM7 for Apo Imp*α* (dotted line), whereas (B) the twisting motion was quantitatively characterized by the average values for the angles between helices along NM9 (solid line) and PC3 (dashed line) for Imp*α* -NplNLS and along NM9 for Apo Imp*α* (dotted line).

The bend motions in NM7/PC1 were characterized by the opening and closing of Imp*α* in the concave surface, along the NLS binding pockets. The quantitative analysis using geometric measurements determined the radius of curvature in opened and closed configurations of Imp*α* (Fig 4A, S12 Fig and S1 Movie) and showed different amplitudes for the bending motion, in which Apo Imp*α* had higher amplitudes than the Imp*α*-NplNLS complex.

**Fig 5:**
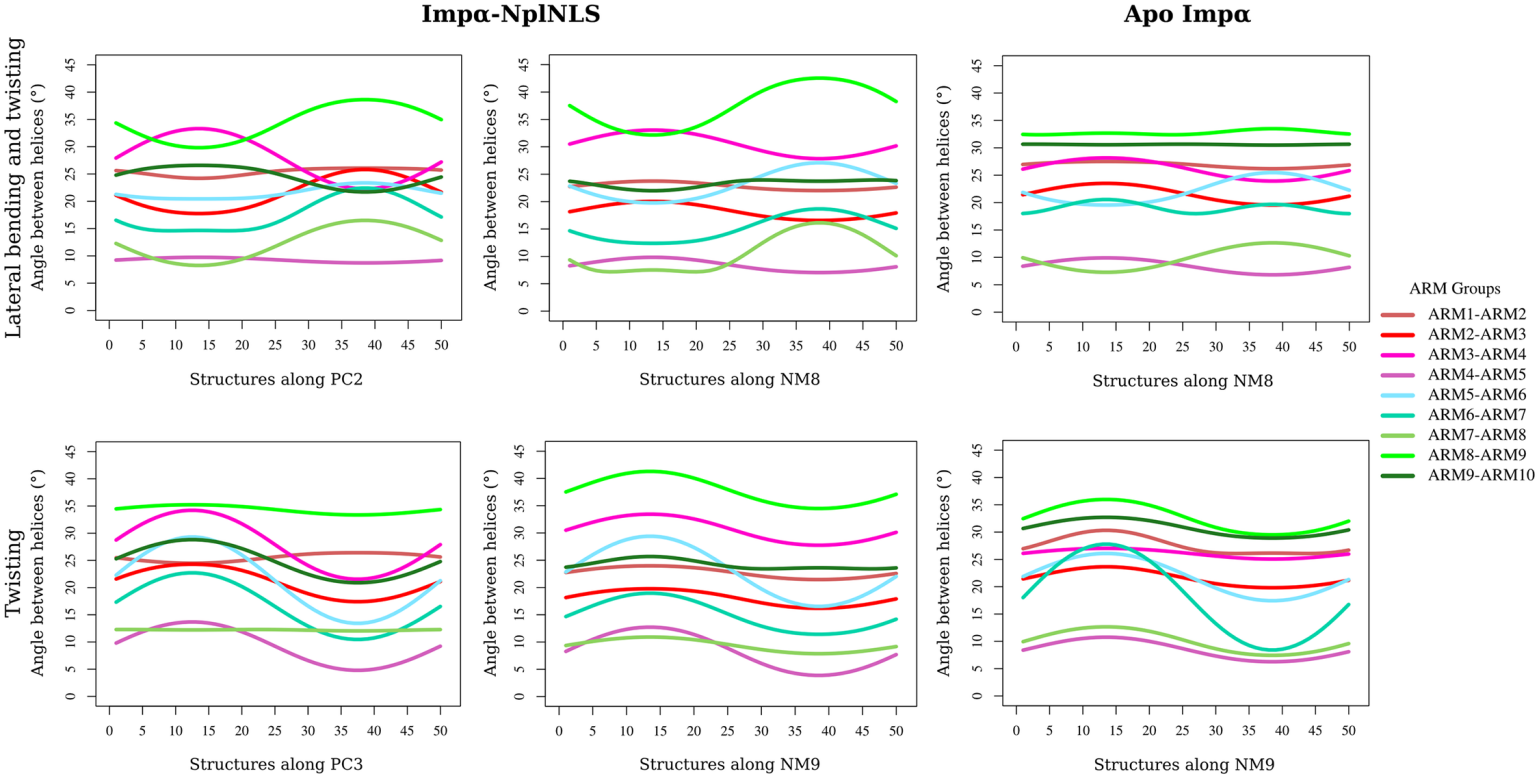
Angle between helices of Imp*α*. The angles between neighboring H3 pairs from the motions described as lateral-bending/twisting (along PC2 and NM8) and twisting (along PC3 and NM9) for Imp*α* -NplNLS and Apo Imp*α*. The ARM groups considered for each angle calculation are depicted with different color assignments.

The twist pattern for the Imp*α* structures along NM9/PC3 (Fig 3, S12 Fig and S3 Movie) oscillated from maximum and minimum values of torsion over the entire protein (Fig 4B). A general observation of the angles for the Imp*α*-NplNLS complex and Apo Imp*α* showed similarities among the structures. A more detailed analysis, considering the inter-repeat angles (Fig 5), detected oscillation between the ARMs in the Imp*α*-NplNLS complex, primarily between ARMs 5–6. Movements with smaller amplitudes were observed for most pairs of ARMs in Apo Imp*α*, except the oscillation observed between ARMs 6–7.

Bending and twisting movements were also evaluated for the crystal structures of Imp*α* in the presence of different types of bipartite cNLSs. A comparison of geometries indicated a small difference among them in the order of tenths of ångströms (S5 Table). The qualitative vector analysis of Imp*α*-NplNLS NM8/PC2 indicated a combination of two motions, characterized by a “lateral” bending tendency (based on a 90° X-axis rotation of Imp*α* in relation to the bending orientation in NM7/PC1) in ARMs 1–6, mixed with a twist in ARMs 7–10 (Fig 3, S12 Fig and S2 Movie). The amplitude of the inter-repeat angle variation was slightly distinct between NM8 and PC2; however, in both cases, we could verify motions that almost reached both sites, including their intermediate region (Fig 5). The complexity of these mixed motions increased in the following modes (S11 and S13 Figs). The same analysis for Apo Imp*α* NM8 showed the reduction of movement amplitude for most ARMs.

### Main contacts in Imp*α*-NplNLS interface

**Fig 6:**
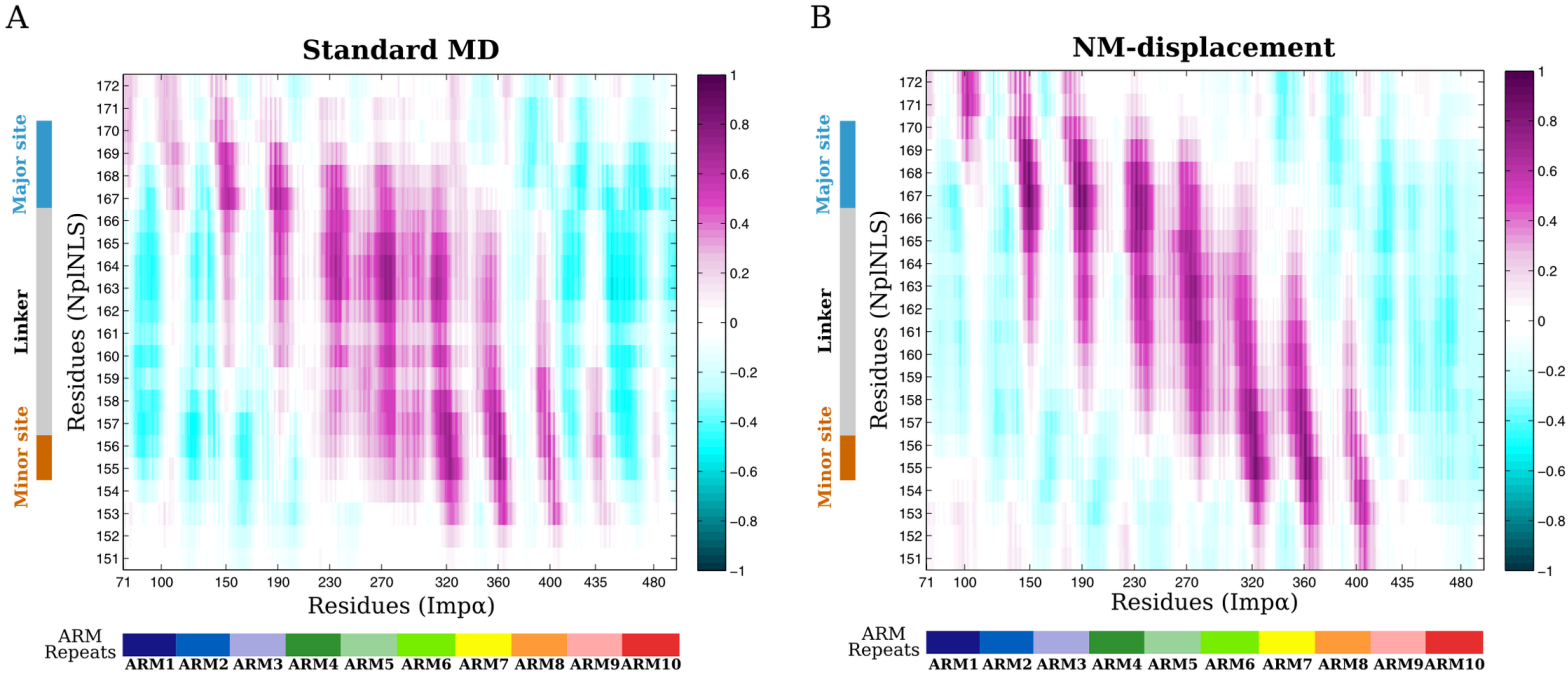
Heatmap of cross-correlations between Imp*α* and NplNLS. (A) Trajectories from standard MD simulations (300 ns ensemble) and (B) NM-displacement (ensemble from references structures 67,730 ps, 207,080 ps and 274,970 ps) were used for the calculation of correlations. A color bar indicates the degree of correlation from anti-correlated (negative values) to correlated (positive values) residues. The X and Y axes, respectively, show the position of each ARM repeat in relation to the Imp*α* sequence and the NplNLS residues in contact with the protein binding sites.

In general, the cross-correlations results from MD and NM analyses were similar (Fig 6). Well-bounded areas of positive correlations could be identified, highlighting the NplNLS range of residues in contact with Imp*α* major and minor sites and the linker region. However, for standard MD, the correlations were more scattered in the linker.

**Fig 7:**
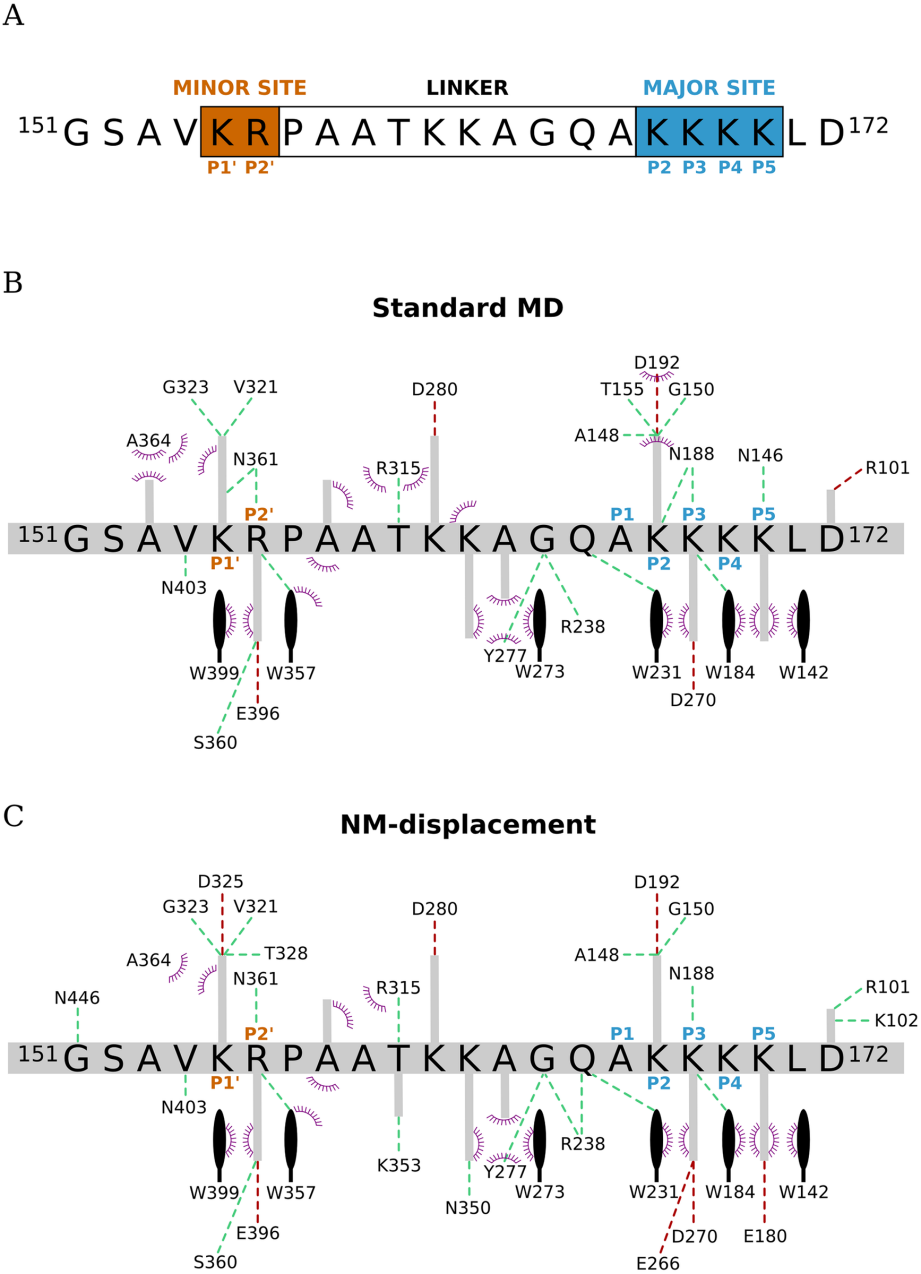
Schematic representation of the interactions observed in the Imp*α*-NplNLS interface. (A) The major (P2–P5) and minor (P1’-P2’) sites and the linker region are indicated in the sequence of the NplNLS. (B) The standard MD (300 ns ensemble) and (C) NM-displacement (ensemble from reference structures 67,730 ps, 207,080 ps and 274,970 ps) interaction scheme is shown with salt bridges (red) and hydrogen bonds (green) as dashed-lines, and hydrophobic contacts are shown as arcs with radiating spokes. The important tryptophan residues that mediate the hydrophobic contacts are depicted in the scheme as black sticks. The main chain of the NplNLS is represented as a gray horizontal line with its respective amino acid sequences, together with side chains shown as perpendicular lines. Only interactions that had an occupancy rate ≥50% of the analyzed trajectories are indicated in the scheme.

Subsequent analysis showed that most correlations could be related to specific interactions in the interface of Imp*α*-NplNLS. Compared to our simulation data, the starting structure exhibited more contacts of hydrogen bonds and hydrophobic interactions (S1 Fig). Throughout the simulations, some of those initial contacts were lost, and MD and NM showed mostly common results (Fig 7). In particular, we observed salt bridges (_Imp*α*_E^396^, _Imp*α*_D^280^, _Imp*α*_D^192^ and _Imp*α*_D^270^) that were established along the NplNLS, specifically in P2’, P2 and the linker region. Hydrophobic contacts mediated by tryptophans (_Imp*α*_W^399^, _Imp*α*_W^357^, _Imp*α*_W^273^, _Imp*α*_W^231^, _Imp*α*_W^184^ and _Imp*α*_W^142^) occurred mostly in major and minor sites, specifically in P2’, P3 and P5. As expected, a great number of hydrogen bonds were established along the NplNLS, such as _Imp*α*_S^360^, _Imp*α*_N^361^, _Imp*α*_G^323^, _Imp*α*_V^321^, _Imp*α*_R^315^, _Imp*α*_Y^277^, _Imp*α*_R^238^, _Imp*α*_A^148^, _Imp*α*_G^150^ and _Imp*α*_N^188^.

Most of the aforementioned interactions occurred with side chains of the charged residues of the NplNLS. Moreover, we highlighted residues _Imp*α*_D^192^, _Imp*α*_E^396^, _Imp*α*_W^184^, _Imp*α*_W^231^, _Imp*α*_W^357^, _Imp*α*_W^399^ and _Imp*α*_Y^277^ that were observed in more than 90% of the analyzed trajectory frames from MD and NM ensembles (S2, S3 and S4 Tables).

## Discussion

### Bending and twisting motions may be directly related to Imp*α* function

Both bending and twisting motions are good candidates to adapt the Imp*α* to the cNLS, because their motion pattern promotes conformational changes in the NLS binding pockets. The combination of these motion patterns appeared to be similarly recurring in other modes, such as NM8. The vectors from other high-collectivity modes, such as NM10–13 and NM17–18, also exhibited these two main motions but in smaller portions of the protein, and they were also associated with undefined types of motions. This finding was expected because modes with higher frequency are normally associated with localized vibrations [56]. Studies involving the protein Imp*β*– a solenoid protein similar to Imp*α* [1, 31, 57]– suggested the importance of bending and twisting motions to generate the flexibility of Imp*β*, allowing it to bind to different types of proteins [31, 58]. In our Imp*α* computational analysis, the described movements could be equally important to adapt to different sizes of cargo proteins and NLSs, enhancing the contacts over the NLS binding site. Although no significant changes were observed by the geometric analysis from the Imp*α* crystallographic structures bound to different types of bipartite NLSs, we must consider that the presence of only a peptide (approximately 20 amino acid length) submitted to similar crystallization conditions may not be sufficient to induce large conformational changes in this system. Therefore, the role of Imp*α* motions is likely critical for the cNLS accommodation, considering the entire protein that contains it.

The MD/NM approaches complementarily showed the flexibility of Imp*α* by the analysis of NM7–9 and PC1–3. The bending observed in NM7/PC1 may be directly related to the accessibility to the binding sites and the release of NLSs. This movement was observed in both Imp*α*-NplNLS and Apo Imp*α*, with small differences in their amplitude, resembling “open” and “close” movements. Bending motions have been reported in globular proteins to identify movements of domains along with opened and closed states [59, 60]. The bending analysis for Imp*α* was possible because its small curvature [1] allowed for the establishment of a plane comprising two distal (N- and C-termini) atoms and a central atom. Solenoid Imp*β* required a more complex analysis, determining angles between vectors projected onto a reference plane in each motif to evaluate the curvature changes [31]. Specifically, the concerted bending motion of Imp*α* NM7 could operate as an opening-closing gateway, allowing the NLS entrance and adjusting the amplitude of this motion in relation to the cargo protein size. The absence of a ligand may imply in a wider curvature favoring the access to the inner concave surface of Imp*α*, allowing the IBB domain or cargo protein binding. Pumroy et al. [61] compared the flexibility of three human Imp*α* isoforms (*α*-1, *α*-3 and *α*-7) considering their bound and unbound states and applying MD simulations. Based on the protein end-to-end distance measurements, the authors observed an increase in the flexibility of Apo Imp*α* isoforms, in accordance with our bending characterization that showed a higher radius of curvature for Apo Imp*α*.

According to Kobe et al. [1], the “twist” takes into account the rotations of the neighboring repeats relative to each other along the backbone direction, and ARM repeat proteins have large twist movements, allowing the accommodation of extended and flexible peptides. The twist observed in the NM9/PC3 of Imp*α*-NplNLS showed similarities to each other, confirming the observations of Hayward et al. [62], which compared NM/PC for lysozyme protein. Although Imp*α*-NplNLS and Apo Imp*α* had similar average values (Fig 4B), the differences were found only in individual analyses of the neighboring ARM repeats. The angle variation between ARM6 and ARM7 in NM9 of Apo Imp*α* (Fig 5) is remarkable, and a similar result was already observed for Apo Imp*α*-3 [61]. The lack of a ligand (IBB domain or NLS) interacting with the Apo Imp*α* concave portion may lead to a higher twist in the middle of Apo Imp*α* due to the lack of interactions that stabilize and provide binding specificity. The increment of the angle amplitude in this region was contrasted by lower values for the remaining protein regions compared to Imp*α*-NplNLS. Therefore, Imp*α*-NplNLS may require conformational changes for the NLS adjustment to the binding pockets of the Imp*α* inner surface, which explains the greater variation in the ARM’s angles in contrast to Apo Imp*α*, which would not require such adjustments. This same explanation applies to the amplitude variations also found in NM8 for the bound/unbound states of Imp*α*.

Experiments and MD simulation with *Neurospora crassa* Imp*α* emphasize the instability of an N-terminally truncated Imp*α* in the absence of an NplNLS peptide, indicating the occupancy of the NLS binding sites as a requirement for Imp*α* crystallization [63]. In addition, Falces et al. [64] also showed, performing circular dichroism assays with Imp*α*-1*Δ*IBB from *Xenopus laevis*, the stabilization of the protein upon association with Npl, thus reinforcing the aforementioned observations. The establishment of polar contacts settles the NLS backbone, whereas hydrophobic and electrostatic interactions with positively charged NLS residues allow the specificity [16, 21]. The greater structural flexibility of Apo Imp*α* was only observed by the geometrical analysis for the NM7 bending. Despite the non-association of the NM8/NM9 twisting motions to a greater overall flexibility of Imp*α*, the NLS absence allowed for a greater motion amplitude of other NMs, such as modes 21–27, comparing the restriction energies of Imp*α*-NplNLS (S4 Fig) and Apo Imp*α* (S5 Fig), indicating that the higher flexibility of Apo Imp*α* appeared to be distributed over these modes. In addition, considering the C*α* fluctuations of Imp*α* in its bound and unbound states (S9 Fig), we observed a higher flexibility in the major site of Apo Imp*α*. This result was also observed for Apo Imp*α*-3 [61], and the authors predicted a weaker binding for NLSs that relies primarily on the major site. However, this statement does not seem to apply to our case because Imp*α*-2 has been crystallized with different types of NLSs, including those with preference to the major site; e.g., simian virus 40 (SV40) NLS [24]. Moreover, the binding stability of Imp*α*-2 to some of those NLSs was also observed with ligand binding assays [17, 65, 66]. In summary, the NLS binding stabilizes Imp*α*, apparently restricting the motion range represented by some higher-frequency NMs; however, it may also allow localized adjustments between the ARM repeats to improve the overall affinity to the bound NLS.

### The role of the linker residues in cNLS recognition

The cross-correlation calculations performed for both MD simulations (Fig 6A) and displacement along NMs (Fig 6B) showed positive correlations from residues of both major and minor sites of Imp*α*. Further analysis showed that the majority of positive correlations observed could be explained by the correspondence of salt bridges, hydrogen bonds and hydrophobic interactions. We could determine that positions P2 and P5 from the major site and P1’ and P2’ of the minor binding site have high levels of contact with the NplNLS, based on the occupancy values of some interactions in those regions. These data confirm the maintenance of the main contacts between Imp*α* and NplNLS, showing that our simulation results are in agreement with the experimental data [17, 24].

A classical bipartite NLS sequence interacts simultaneously with major and minor binding sites of Imp*α* and depends on a linker region containing a minimum of 10 residues between P2’ and P2 positions to act as a cNLS [5, 30]. The linker region in the NplNLS appears to play a key role in the process of cNLS recognition. The cross-correlation maps clearly indicated an involvement of this region in the promotion of contacts with Imp*α*. A detailed analysis of the interactions showed the occurrence of salt bridges, hydrogen bonds and hydrophobic contacts along the NplNLS. The involvement of _Imp*α*_R^238^, _Imp*α*_R^315^, _Imp*α*_W^273^ and _Imp*α*_Y^277^ was already observed in Imp*α*-NplNLS crystal structures [17, 24]. However, the computational approach indicated other interactions previously reported in single structures; _Imp*α*_K^353^ and _Imp*α*_N^350^ were reported with the NLS from FEN-1 [30] and _Imp*α*_D^280^ from SV40 NLS bound to the Imp*α* from a filamentous fungus [67]. Moreover, new possible contacts were detected; e.g., residues _Imp*α*_N^446^, _Imp*α*_R^101^ and _Imp*α*_R^102^ interacted in the N- and C-terminal regions of the NLS.

Our simulation analyses suggest that the linker contacts are important to settle the cNLS, and help to accommodate the cNLS side chains into the grooves of major and minor binding sites. The linker region, in addition to the N- and C-terminals of an NLS, may also compensate for interactions for the establishment of an activation pattern of the other NLS region [22], which indicates that the linker contacts may occur in different NLSs and can be maintained after the docking of the bipartite NLS in both major and minor binding sites, depending on the residues composing the linker, such as proline and acidic amino acids [5, 22]. In summary, the recurrent presence of residues outside the major and minor sites strongly reinforces their importance for the proper binding of the NLS, which corroborates other studies involving other NLSs [21, 25–29] and further encourages us to understand in greater detail their roles and effects during the nuclear import process.

### NM analysis with classic MD simulations

MD simulations and NM analysis have been used for macromolecules as a complementary analysis to the experimental data to describe their main motions and relate to a specific function [59, 60, 68, 69]. The approach used for the Imp*α*-cNLS complex combines NMs robustness—to evaluate wider protein movements—with the reference structures from the MD and indicates the benefits of this association in protein-peptide analysis.

The data sampling from MD simulations, despite the time constraints and convergence difficulties associated with this technique [70, 71], were balanced among the selected reference structures. Considering the time of the simulations, there is a likely stability of the studied system. Although we have not performed classical MD of Apo Imp*α*, Takeda et al. [63] reinforce the instability of an N-terminally truncated Imp*α* in the absence of an NLS peptide. One way of computationally evaluating the N-terminally truncated Imp*α* would be applying long MDs to observe the effects of high protein flexibility. However, with a simple NM calculation, we could observe in general, based on the restraint energy profile, RMSF values and geometrical analysis, the higher flexibility of Imp*α* in the absence of the NLS.

The MD data with the Imp*α*-NplNLS complex not only supported the maintenance of the protein-peptide complex but also showed some of the major movements and interactions occurring at the complex interface. NMs promoted an analytical method to access the dynamics of the system, allowing the possibility of the recognition of new interactions and dribbling the convergence and conformational restrictions from standard MD. The main motions described here in NMs were also obtained in the PC analysis, in agreement with the comparison between the lowest NMs and the first PCs obtained from MD simulations in the lysozyme model [62] and a subunit of the GroEl chaperone [69]. The highlighted movements are of high occurrence in the protein’s lifetime and are likely to be functionally important. Moreover, the low computational cost of NM is once more an attractive feature for application in biological systems.

## Conclusions

Computational approaches of MD and NM analysis were combined to evaluate the main of motions of Imp*α* and its interaction to a cNLS peptide. The bending motion may be involved in the NLS entrance and the accommodation of cargo protein depending on its size, whereas the twist motions may be involved in the NLS recognition and accommodation into the Imp*α* binding sites. The combination of these movements could allow local adjustments between the ARM repeats, which could improve the overall affinity to the cNLS. The absence of an NLS was also evaluated and may imply in a wider curvature of Apo Imp*α*, allowing for the IBB domain or cargo protein binding. Moreover, a higher twist in the middle of Apo Imp*α* was detected possibly due to the lack of interactions that stabilize and provide binding specificity, which could explain the challenges in crystallizing N-terminally truncated Apo Imp*α*.

The evaluation of salt bridges, hydrogen bonds and hydrophobic interactions corroborates the fundamental interactions between Imp*α* and NplNLS and gives additional support for interactions outside the classical binding pockets that are important during this process. The linker contacts in cNLS assist the adjustment of the peptide backbone, which helps the interactions between cNLS side chains and residues from major and minor binding grooves. In conclusion, MD simulations combined to NM analysis supported the maintenance of the Imp*α*-NplNLS complex exploring the conformational space and accessing the dynamics of the system with a lower computational cost. This approach may help to understand the affinities between Imp*α* and cNLS peptides and non-classic NLSs.

## Supporting Information

S1 Movie Movie of NM7. Imp*α* is shown in a cartoon diagram, and it is colored according to each ARM repeat, from the N-terminal (blue) to the C-terminal (red) ends of the protein. The NplNLS is represented in a cyan cartoon diagram positioned in an antiparallel configuration compared to Imp*α*.
(MP4)

S2 Movie Movie of NM8. Imp*α* is shown in a cartoon diagram, and it is colored according to each ARM repeat, from the N-terminal (blue) to the C-terminal (red) ends of the protein. The NplNLS is represented in a cyan cartoon diagram positioned in an antiparallel configuration compared to Imp*α*.
(MP4)

S3 Movie Movie of NM9. Imp*α* is shown in a cartoon diagram, and it is colored according to each ARM repeat, from the N-terminal (blue) to the C-terminal (red) ends of the protein. The NplNLS is represented in a cyan cartoon diagram positioned in an antiparallel configuration compared to Imp*α*.
(MP4)

S1 Fig Interactions from the starting structure. Scheme of interactions of the starting structure for MD simulations. The representation is similar to the description of Fig 7.
(PDF)

S2 Fig Convergence analysis. Bar plot indicating the frequency of structures clustered within each reference (67,730 ps, 207,080 ps and 274,970 ps) from the MD simulations.
(PDF)

S3 Fig Structural alignment of NplNLS. Structural representation of the NplNLSs from reference structures 67,730 ps (orange), 207,080 ps (green) and 274,970 ps (blue) aligned with the NplNLS from X-ray (red). (A) Stick diagram of the main chain, with major and minor sites indicated. (B-C) Stick diagram including side chains and the residues from each site.
(PDF)

S4 Fig Heatmap of the total restraint energy values from Imp*α* -NplNLS. The values are from the structures generated from the NM-displacement of the most representative reference structure (207,080 ps) according to the convergence analysis. The X-axis is the displacement range, represented as values of mass-weighted root mean square (MRMS), and the Y-axis is the NM numbers. Lower values of energy (blue tons) indicate favorable conformations.
(PDF)

S5 Fig Heatmap of the total restraint energy values from Apo Imp*α*. The values are from the structures generated from the NM-displacement. The X-axis is the displacement range, represented as values of MRMS, and the Y-axis is the NM numbers. Lower values of energy (blue tons) indicate favorable conformations.
(PDF)

S6 Fig Conformational exploration. Box-plot of the RMSD distribution from the trajectories of standard MD (MD-1, MD-2 and MD-3) and NM-displacement (67,730 ps, 207,080 ps and 274,970 ps).
(PDF)

S7 Fig Residue fluctuations of Imp*α* -NplNLS. Residue fluctuations based on C*α* RMSF of Imp*α* from the ensemble trajectories of standard MD (red) and NM-displacement (cyan).
(PDF)

S8 Fig Residue fluctuations of NplNLS. Residue fluctuations based on C*α* RMSF of NplNLS from the ensemble trajectories of standard MD (red) and NM-displacement (cyan).
(PDF)

S9 Fig Residue fluctuations of apo Imp*αΔ*IBB. Residue fluctuations based on C*α* RMSF of Imp*α* from the trajectories of the NM-displacement of Imp*α* -NplNLS (from reference structure 207,080 ps; cyan) and Apo Imp*αΔ*IBB (black).
(PDF)

S10 Fig Collectivity from Imp*α* -NplNLS. The collectivity values are plotted for each NM, and a smoothed line is fitted (blue line), representing the data tendency. The shaded area is the confidence interval around the smoothed line calculated with the ggplot package (http://ggplot2.org/) in R.
(PDF)

S11 Fig Main motions observed in NMs10–13, NM17 and NM18. The vector arrows indicating the motions are shown. The Imp*α* is displayed in a cartoon diagram, with each ARM colored from blue to red, corresponding to N to C-terminals. The NplNLS (cyan) is in a cartoon representation and is positioned in an antiparallel configuration compared to Imp*α*.
(PDF)

S12 Fig Main motions observed in PCs1–3 from standard MD. The vector arrows indicating the motions are shown. The Imp*α* is displayed in a C*α* representation, with each ARM colored from blue to red, corresponding to N to C-terminals. The NplNLS (cyan) is in C*α* representation and is positioned in an antiparallel configuration compared to Imp*α*.
(PDF)

S13 Fig Angles between helices of Imp*α* -NplNLS for NMs10–13, NM17 and NM18. The ARM groups considered for each angle calculation are depicted with different color assignments, similar to Fig 5.
(PDF)

S1 Table Backbone RMSD values from structural alignment. The reference structures are aligned with the Imp*α* -NplNLS crystallographic structure (PDB ID 3UL1).
(PDF)

S2 Table Salt bridges occupancies. The occupancies of salt bridges between NplNLS and Imp*α* in standard MD and NM-displacement. Interactions that were above 50% of occupancy are highlighted in gray.
(PDF)

S3 Table Hydrogen bonds occupancies. The occupancies of hydrogen bonds between NplNLS (blue) and Imp*α* (green) in standard MD and NM-displacement. Interactions that were above 50% of occupancy are highlighted in gray.
(PDF)

S4 Table Hydrophobic contacts occupancies. The occupancies of hydrophobic contacts between NplNLS and Imp*α* in standard MD and NM-displacement. Interactions that were above 50% of occupancy are highlighted in gray.
(PDF)

S5 Table Radius of curvature and average angles for the crystallographic structures. PDB IDs: 1pjm, 1pjn, 3tpm, 3ukw, 3ukx, 3uky, 3ukz, 3ul1 and 3uvu.
(PDF)
