# Supplementary figures and images for: Bending-Twisting Motions and Main Interactions in Nucleoplasmin Nuclear Import
Source: PLoS One. 2016 Jun 3;11(6):e0157162. doi: 10.1371/journal.pone.0157162 (PMC4892583; doi:10.1371/journal.pone.0157162)

# Initial structure

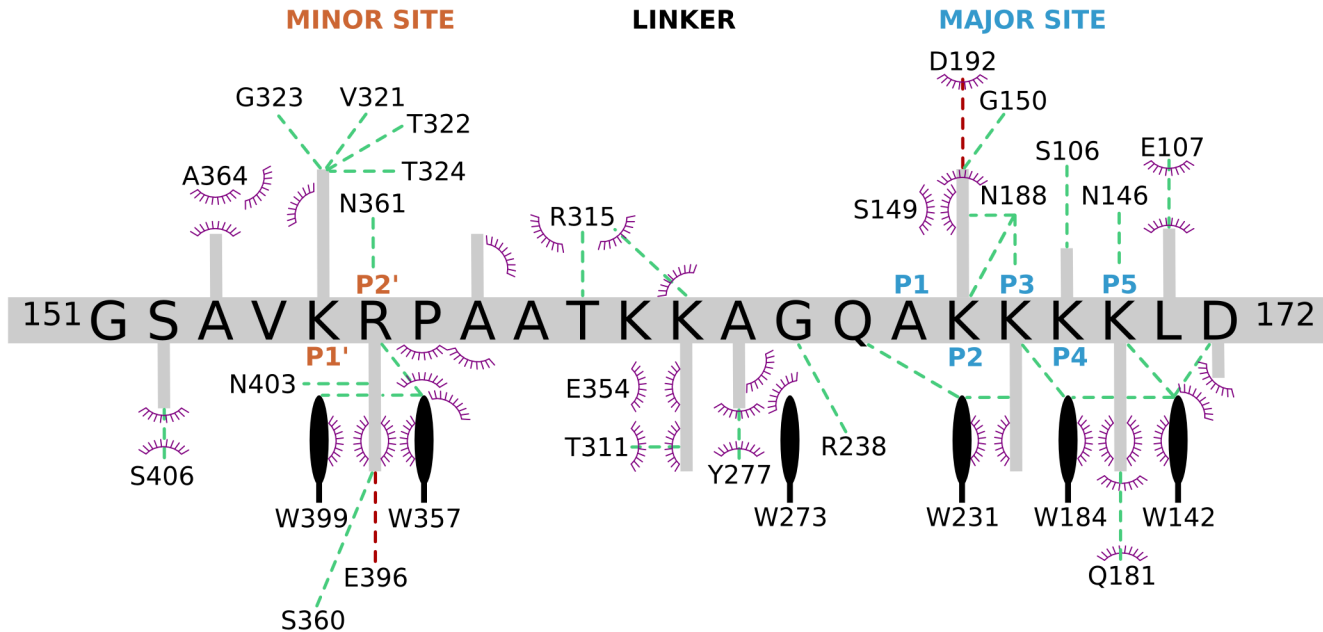

Supplement: S1 Fig — Scheme of interactions of the starting structure for MD simulations. The representation is similar to the description of Fig 7. (PDF) [file pone.0157162.s004.pdf]

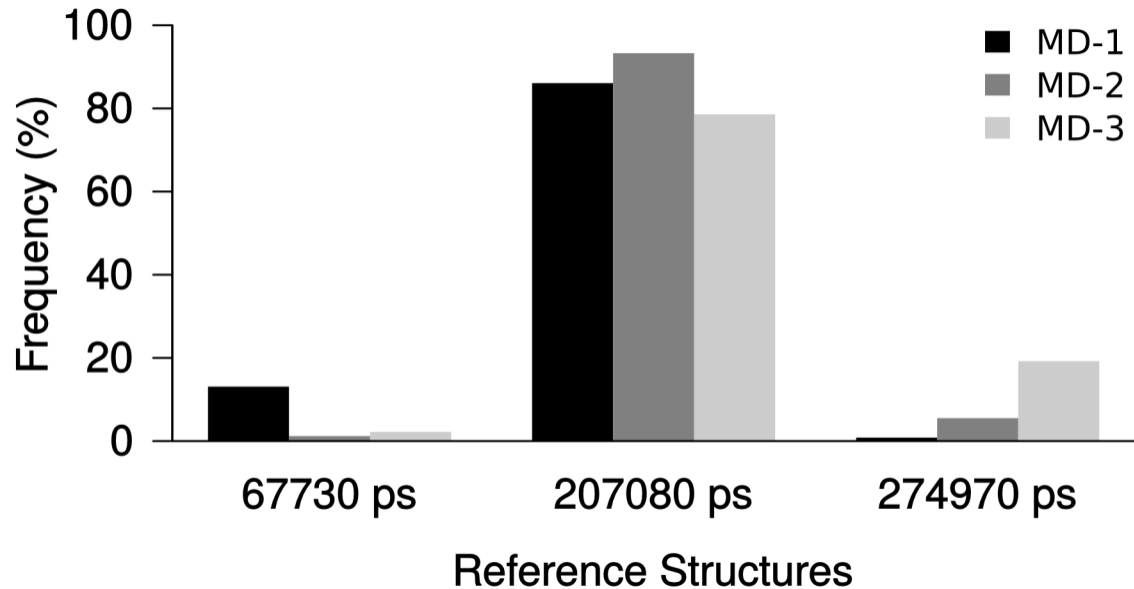

Supplement: S2 Fig — Bar plot indicating the frequency of structures clustered within each reference (67,730 ps, 207,080 ps and 274,970 ps) from the MD simulations. (PDF) [file pone.0157162.s005.pdf]

A

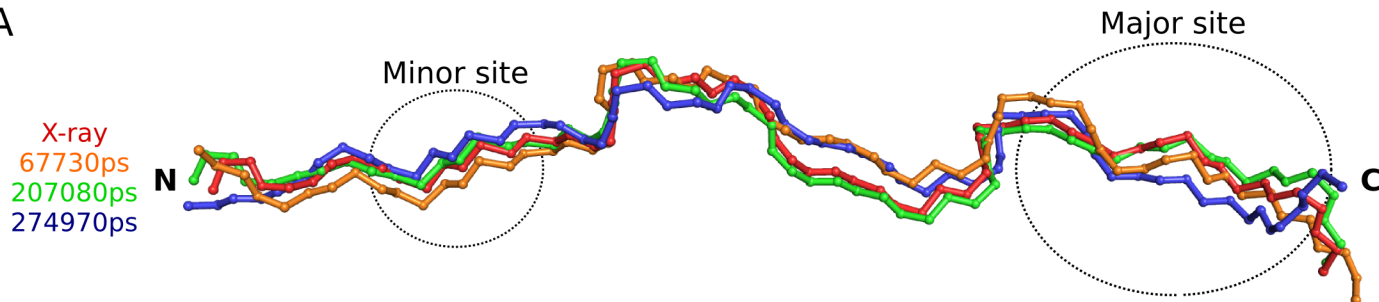

B

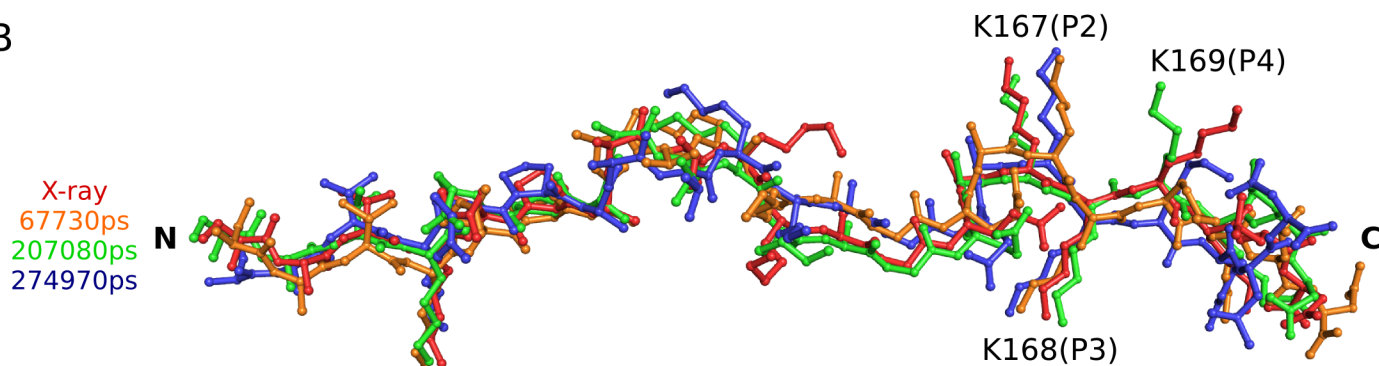

C

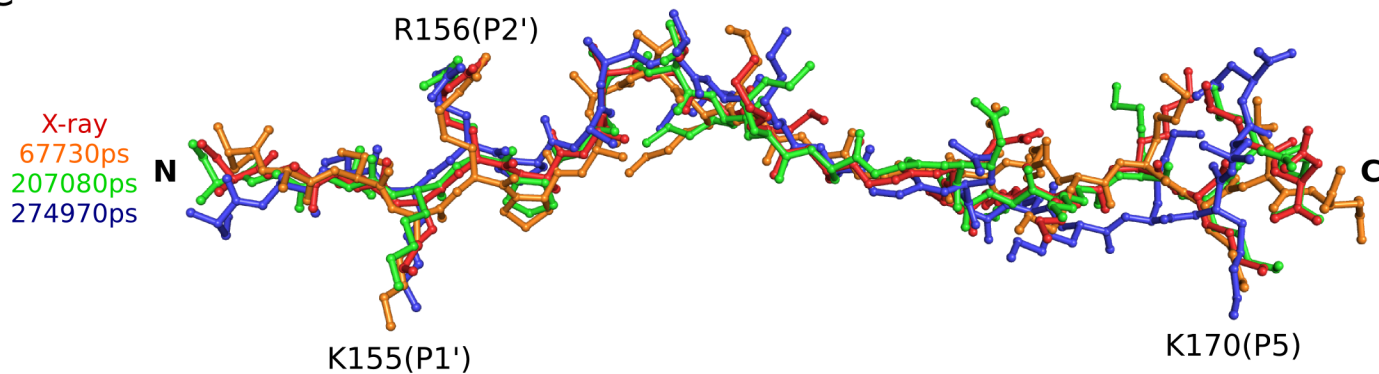

Supplement: S3 Fig — Structural representation of the NplNLSs from reference structures 67,730 ps (orange), 207,080 ps (green) and 274,970 ps (blue) aligned with the NplNLS from X-ray (red). (A) Stick diagram of the main chain, with major and minor sites indicated. (B-C) Stick diagram including side chains and the residues from each site. (PDF) [file pone.0157162.s006.pdf]

# Imp $\alpha$ -NpINLS

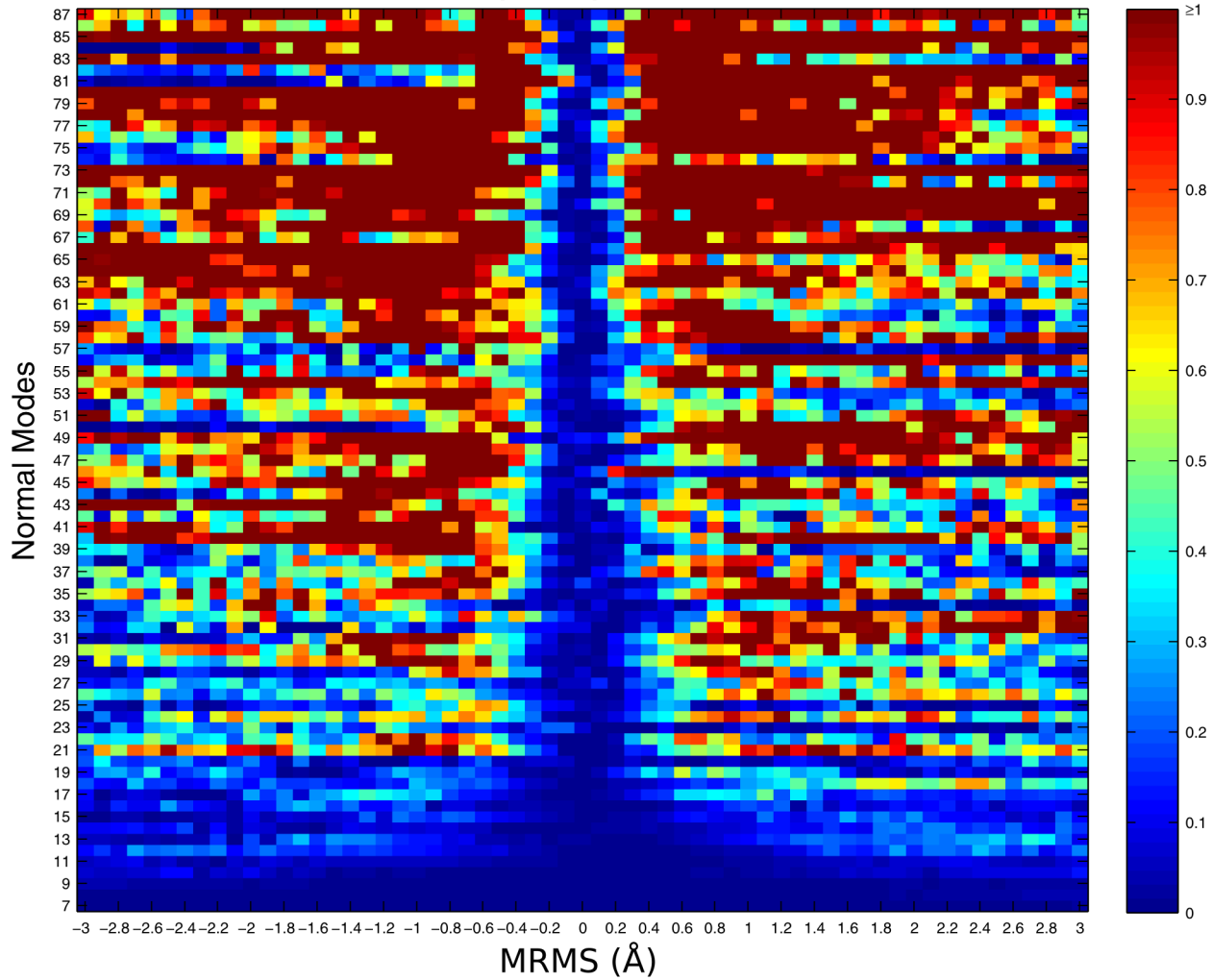

Supplement: S4 Fig — The values are from the structures generated from the NM-displacement of the most representative reference structure (207,080 ps) according to the convergence analysis. The X-axis is the displacement range, represented as values of mass-weighted root mean square (MRMS), and the Y-axis is the NM numbers. Lower values of energy (blue tons) indicate favorable conformations. (PDF) [file pone.0157162.s007.pdf]

# Apo Imp $\alpha$

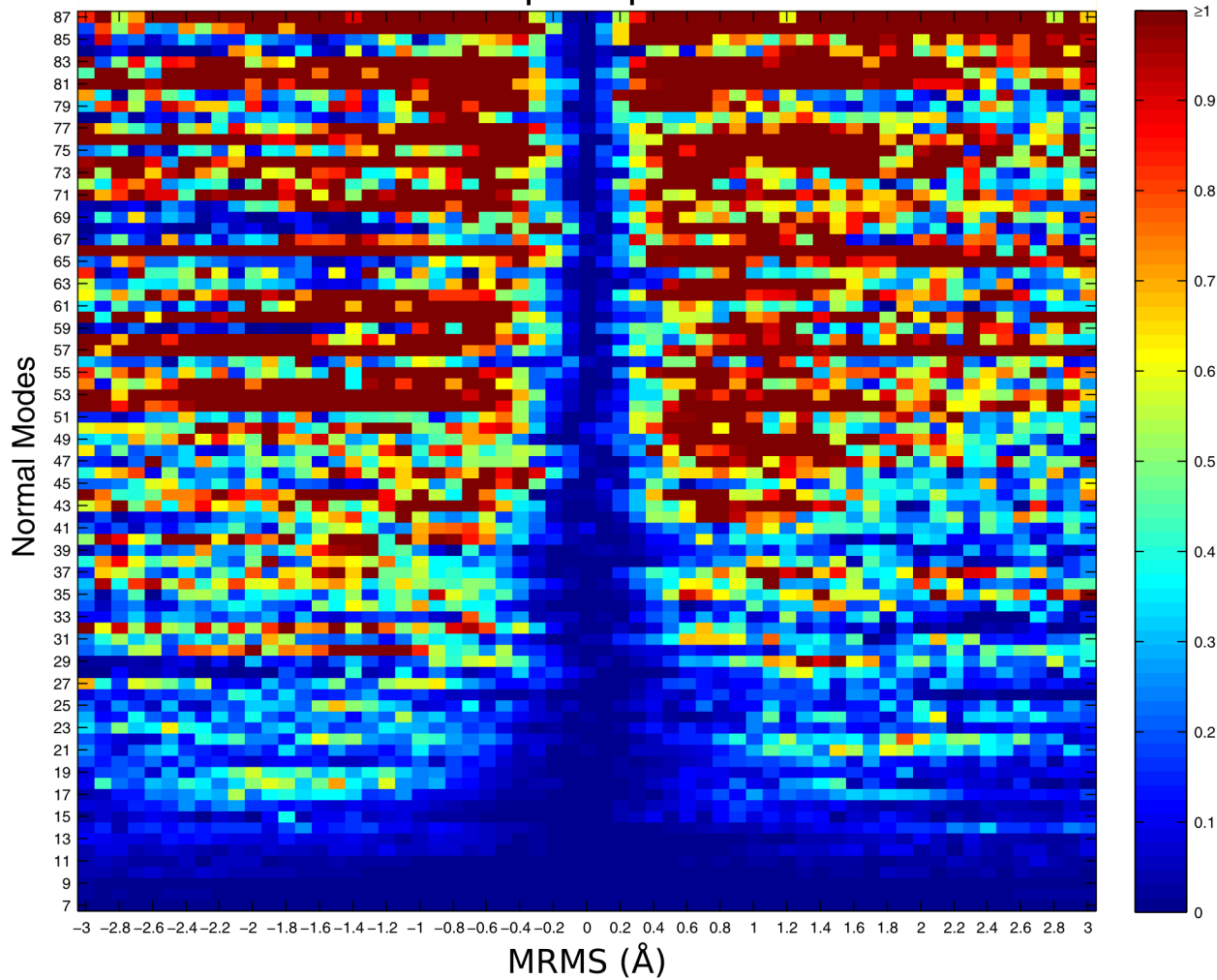

Supplement: S5 Fig — The values are from the structures generated from the NM-displacement. The X-axis is the displacement range, represented as values of MRMS, and the Y-axis is the NM numbers. Lower values of energy (blue tons) indicate favorable conformations. (PDF) [file pone.0157162.s008.pdf]

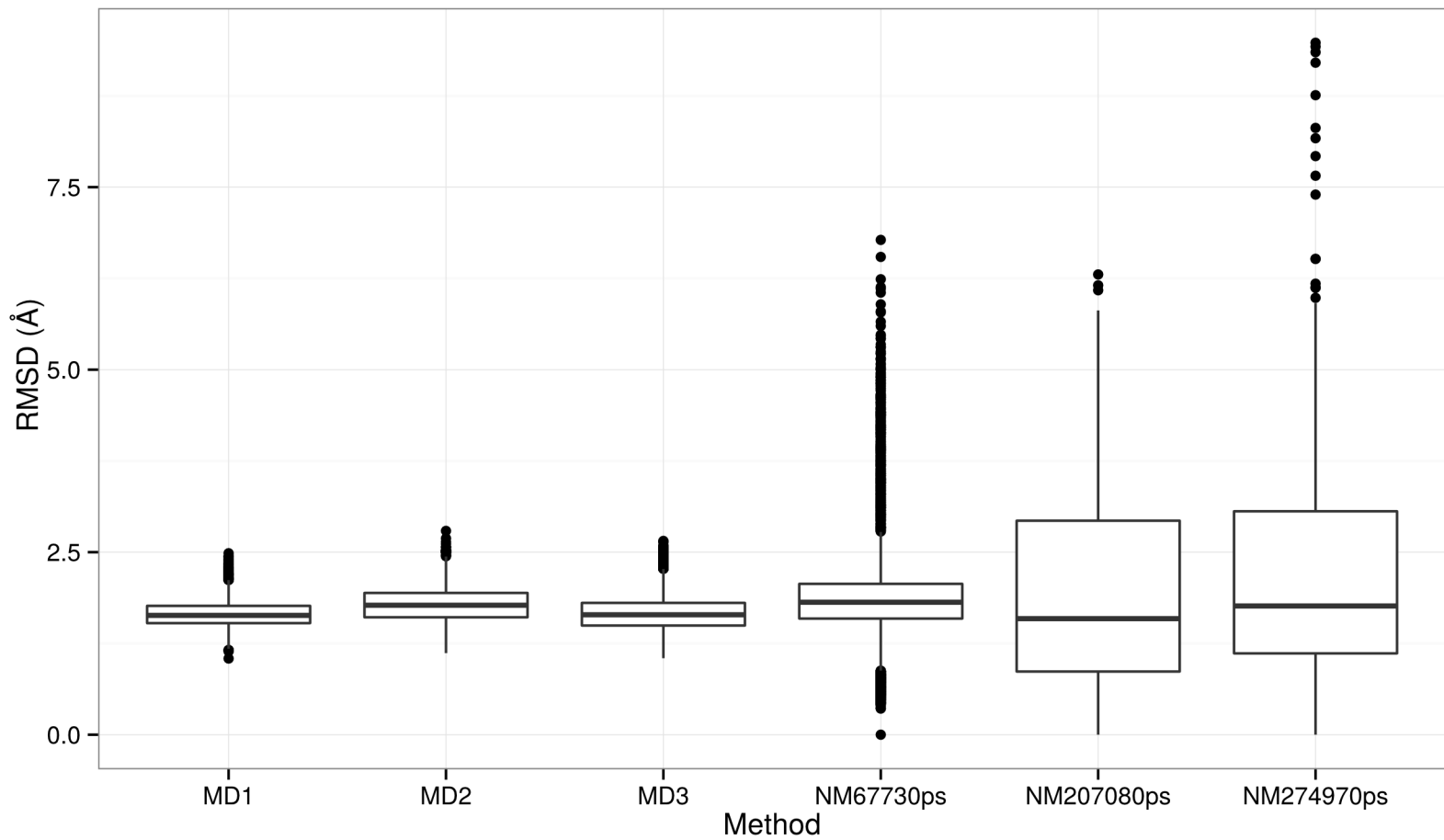

Supplement: S6 Fig — Box-plot of the RMSD distribution from the trajectories of standard MD (MD-1, MD-2 and MD-3) and NM-displacement (67,730 ps, 207,080 ps and 274,970 ps). (PDF) [file pone.0157162.s009.pdf]

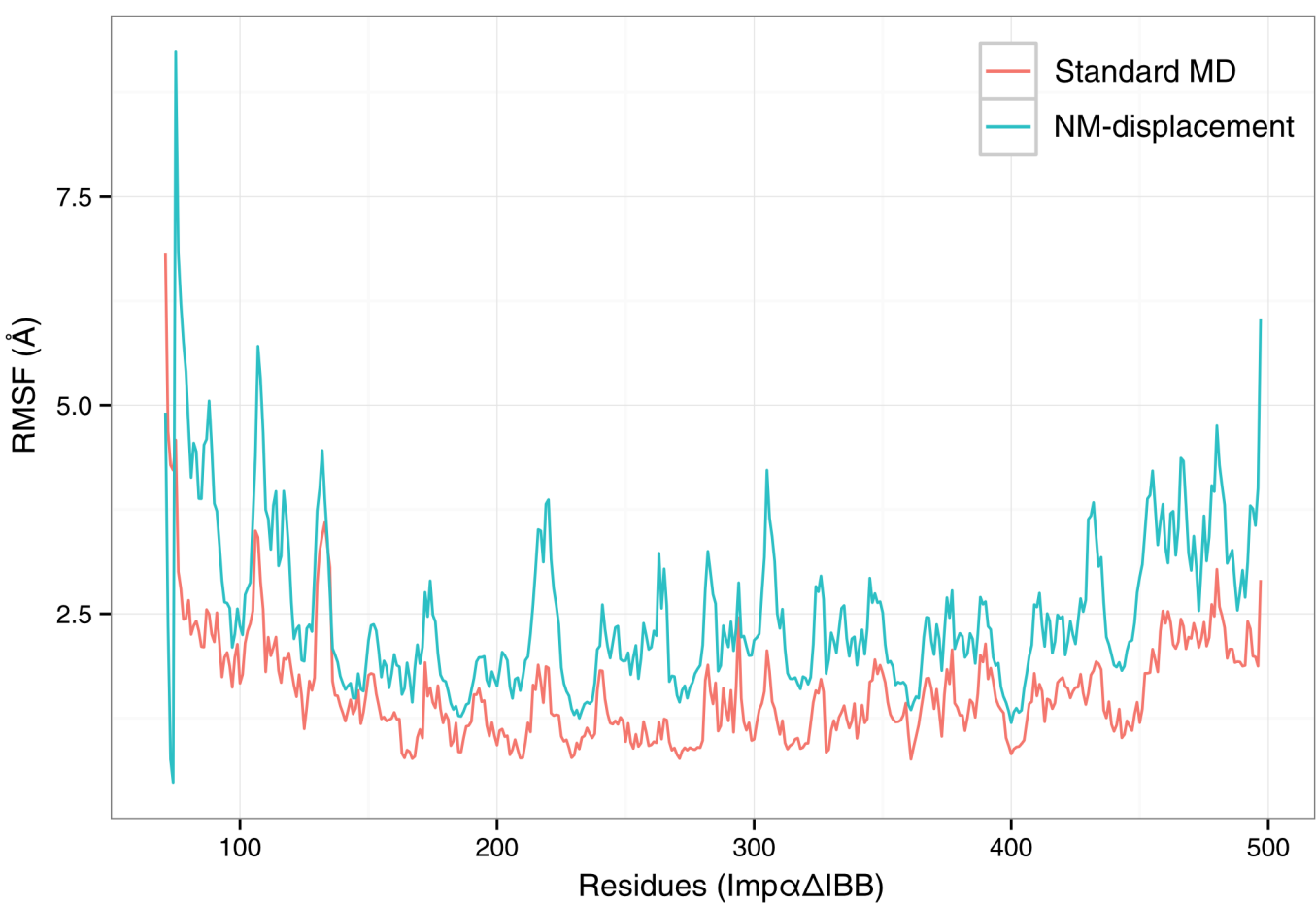

Supplement: S7 Fig — Residue fluctuations based on Cα RMSF of Impα from the ensemble trajectories of standard MD (red) and NM-displacement (cyan). (PDF) [file pone.0157162.s010.pdf]

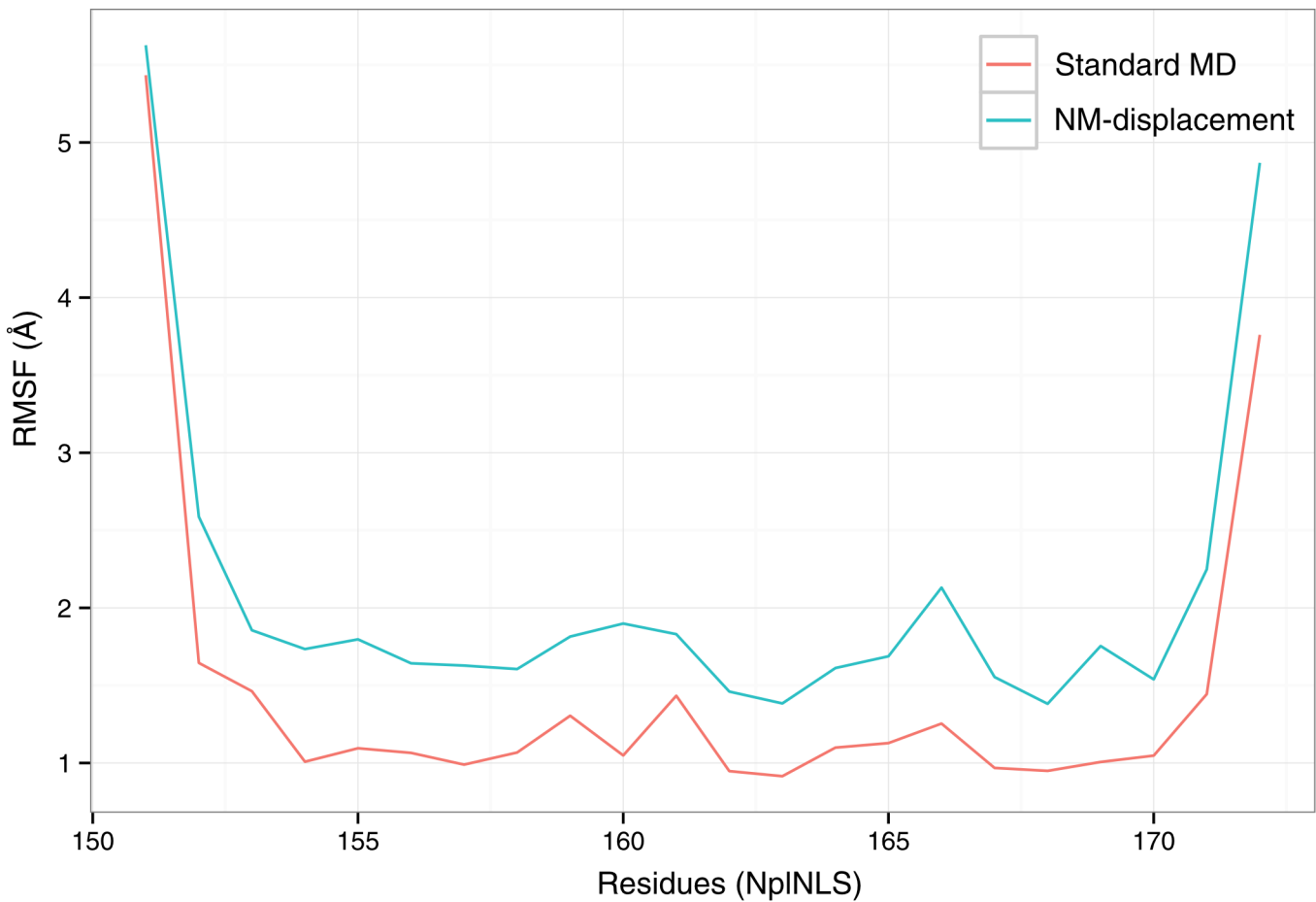

Supplement: S8 Fig — Residue fluctuations based on Cα RMSF of NplNLS from the ensemble trajectories of standard MD (red) and NM-displacement (cyan). (PDF) [file pone.0157162.s011.pdf]

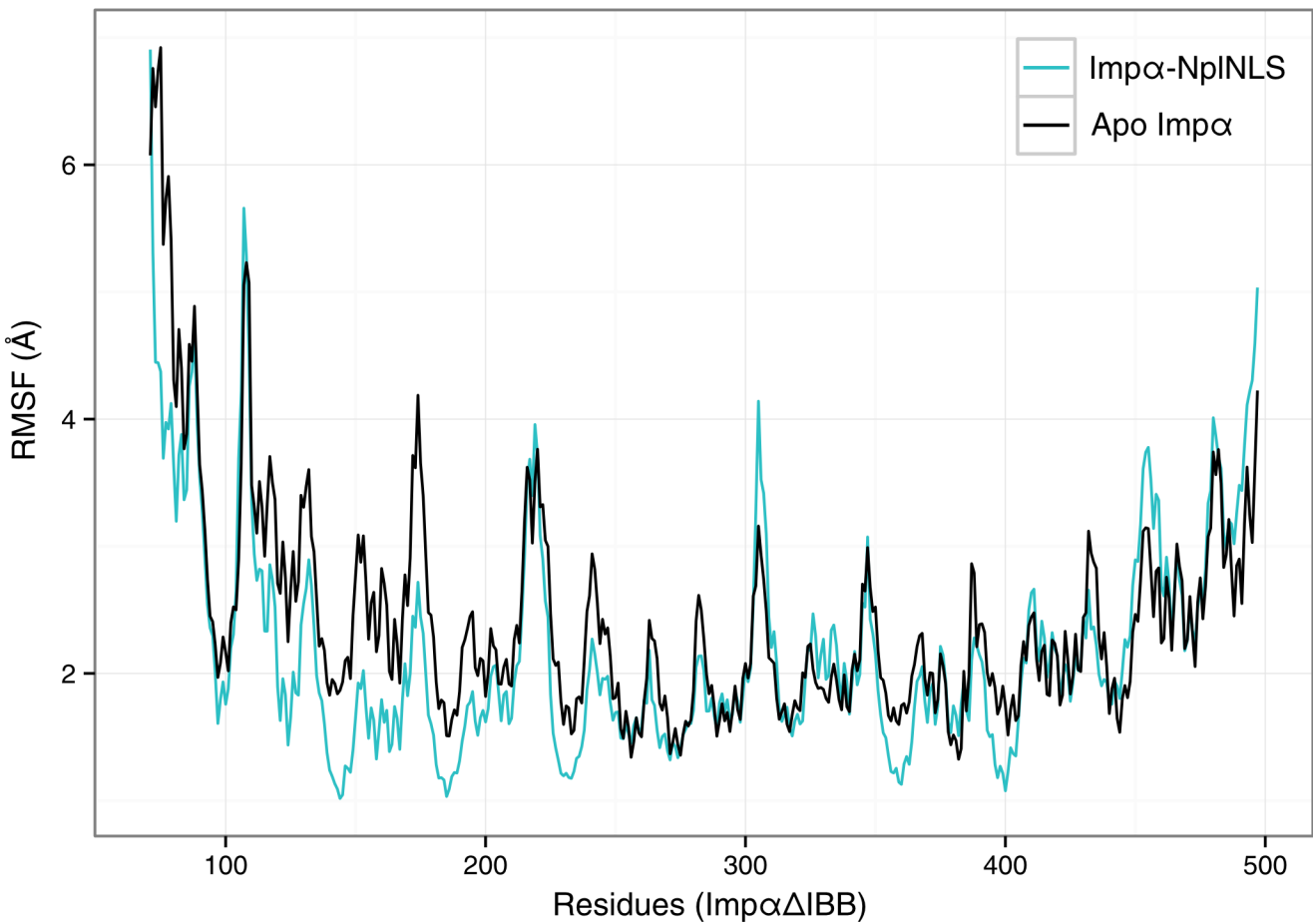

Supplement: S9 Fig — Residue fluctuations based on Cα RMSF of Impα from the trajectories of the NM-displacement of Impα -NplNLS (from reference structure 207,080 ps; cyan) and Apo ImpαΔIBB (black). (PDF) [file pone.0157162.s012.pdf]

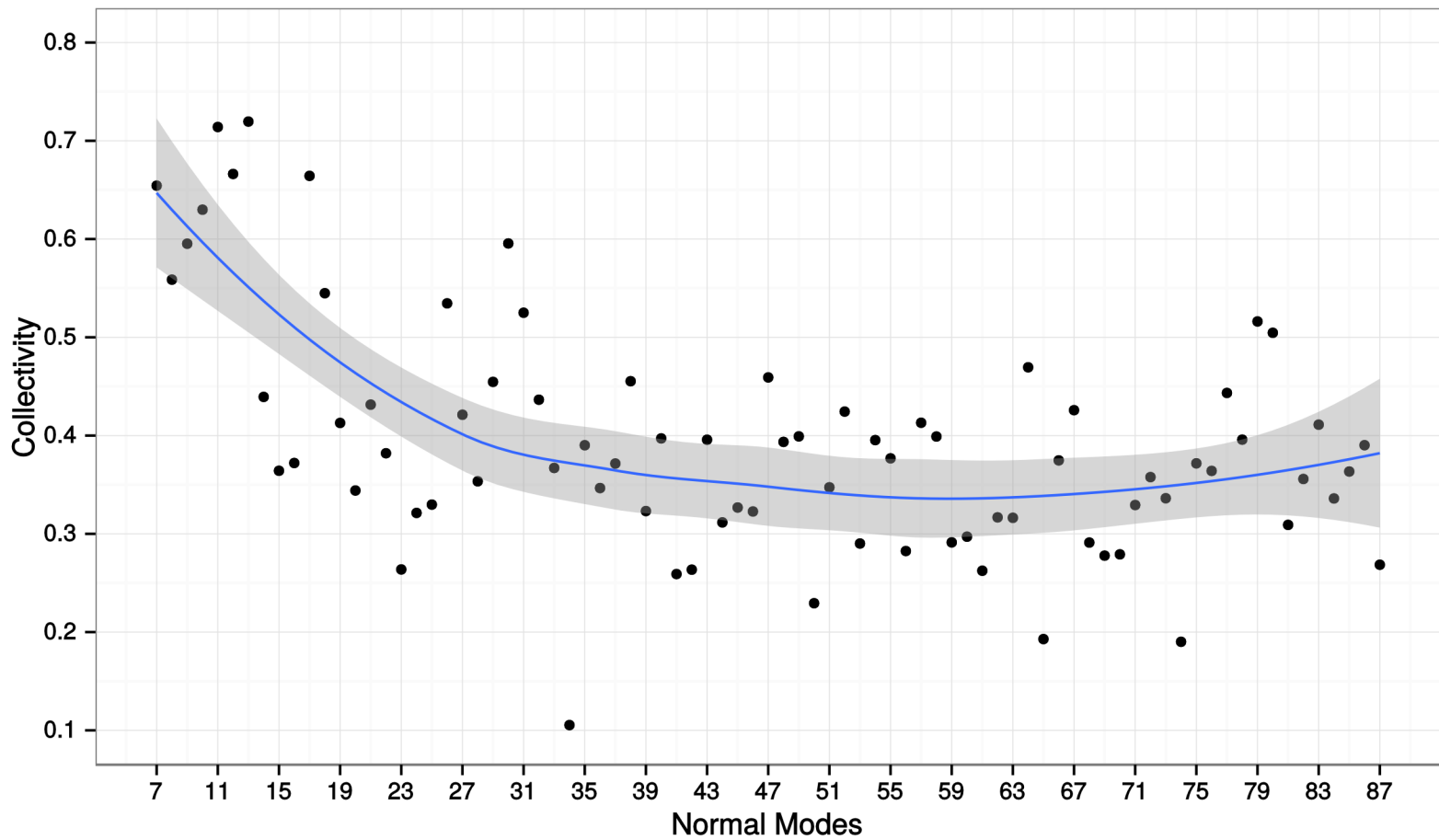

Supplement: S10 Fig — The collectivity values are plotted for each NM, and a smoothed line is fitted (blue line), representing the data tendency. The shaded area is the confidence interval around the smoothed line calculated with the ggplot package (http://ggplot2.org/) in R. (PDF) [file pone.0157162.s013.pdf]

NM10

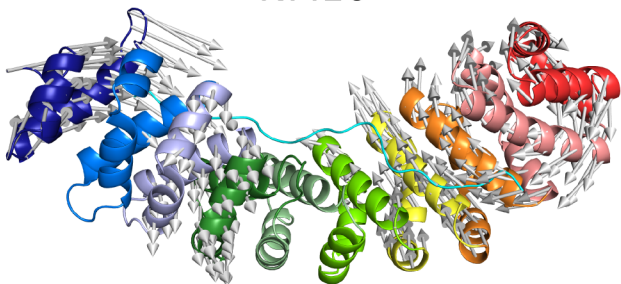

NM11

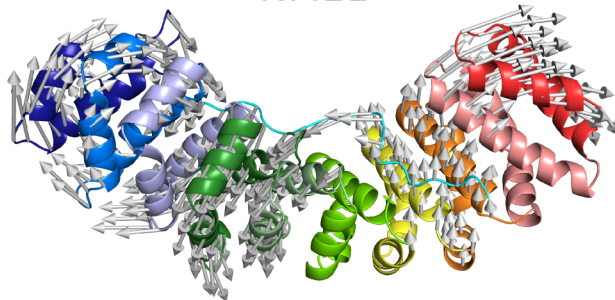

NM12

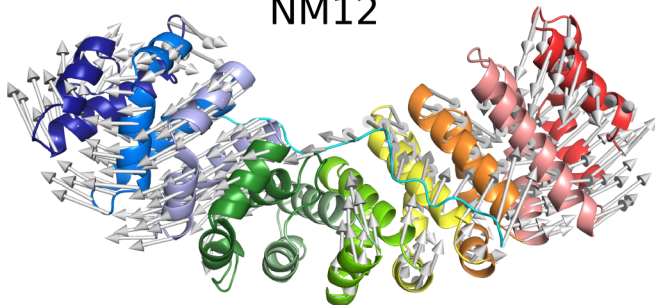

NM13

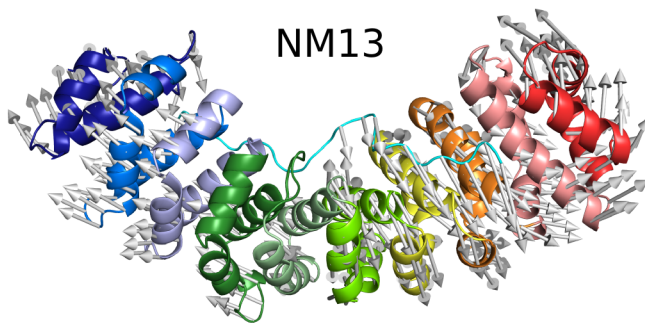

NM17

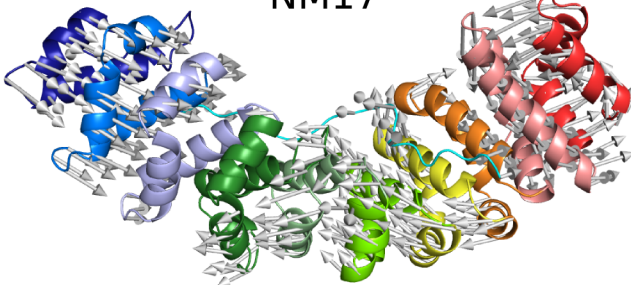

NM18

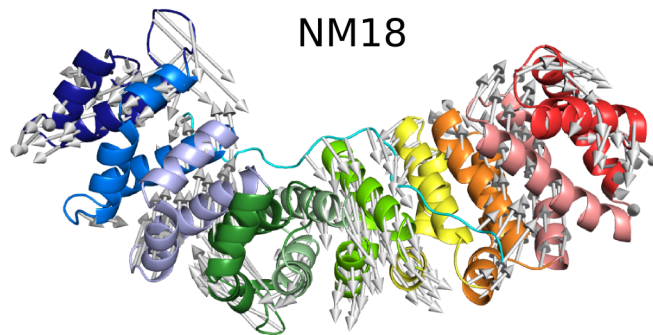

Supplement: S11 Fig — The vector arrows indicating the motions are shown. The Impα is displayed in a cartoon diagram, with each ARM colored from blue to red, corresponding to N to C-terminals. The NplNLS (cyan) is in a cartoon representation and is positioned in an antiparallel configuration compared to Impα. (PDF) [file pone.0157162.s014.pdf]

Bending

PC1

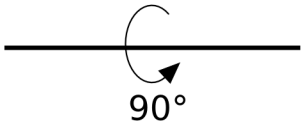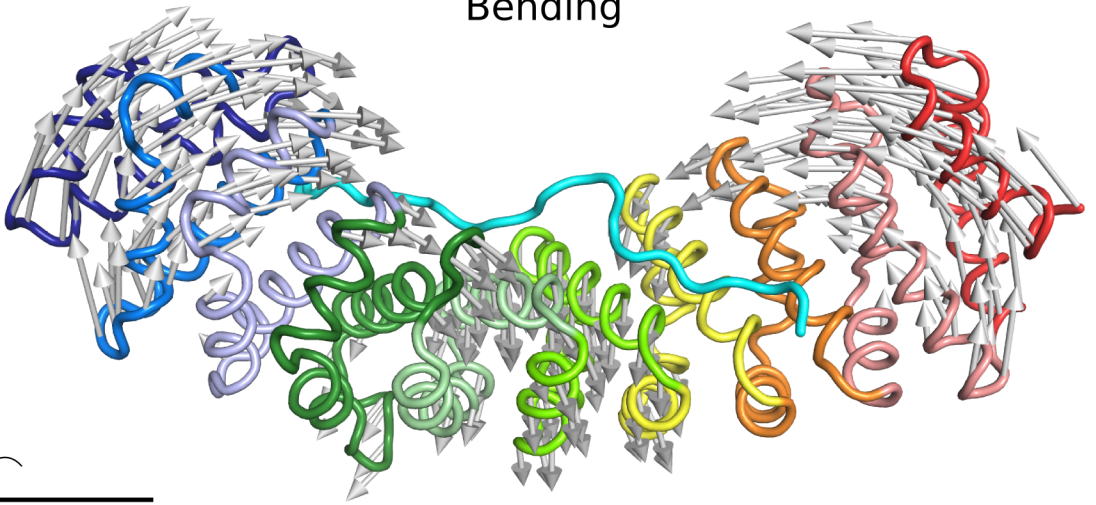

Lateral bending and twisting

PC2

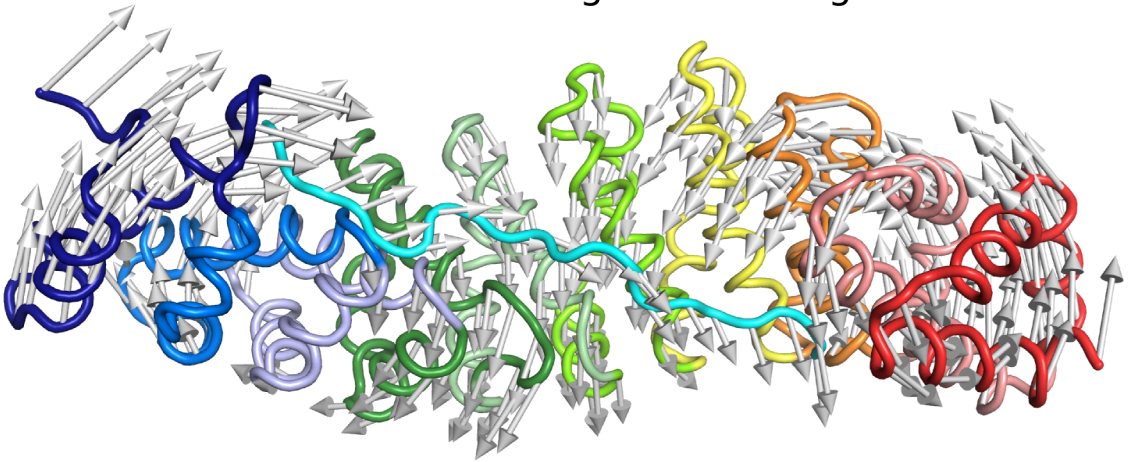

Twisting

PC3

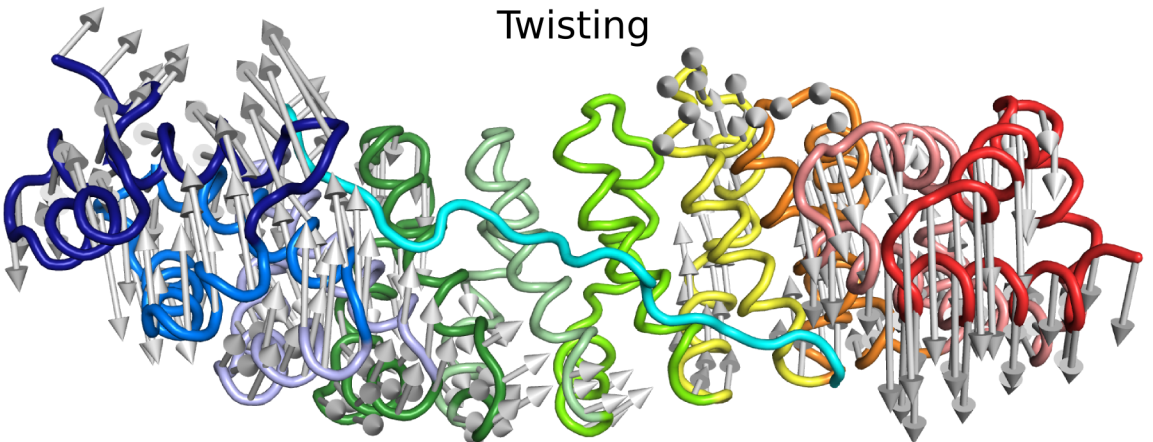

Supplement: S12 Fig — The vector arrows indicating the motions are shown. The Impα is displayed in a Cα representation, with each ARM colored from blue to red, corresponding to N to C-terminals. The NplNLS (cyan) is in Cα representation and is positioned in an antiparallel configuration compared to Impα. (PDF) [file pone.0157162.s015.pdf]

# Imp $\alpha$ -NplNLS

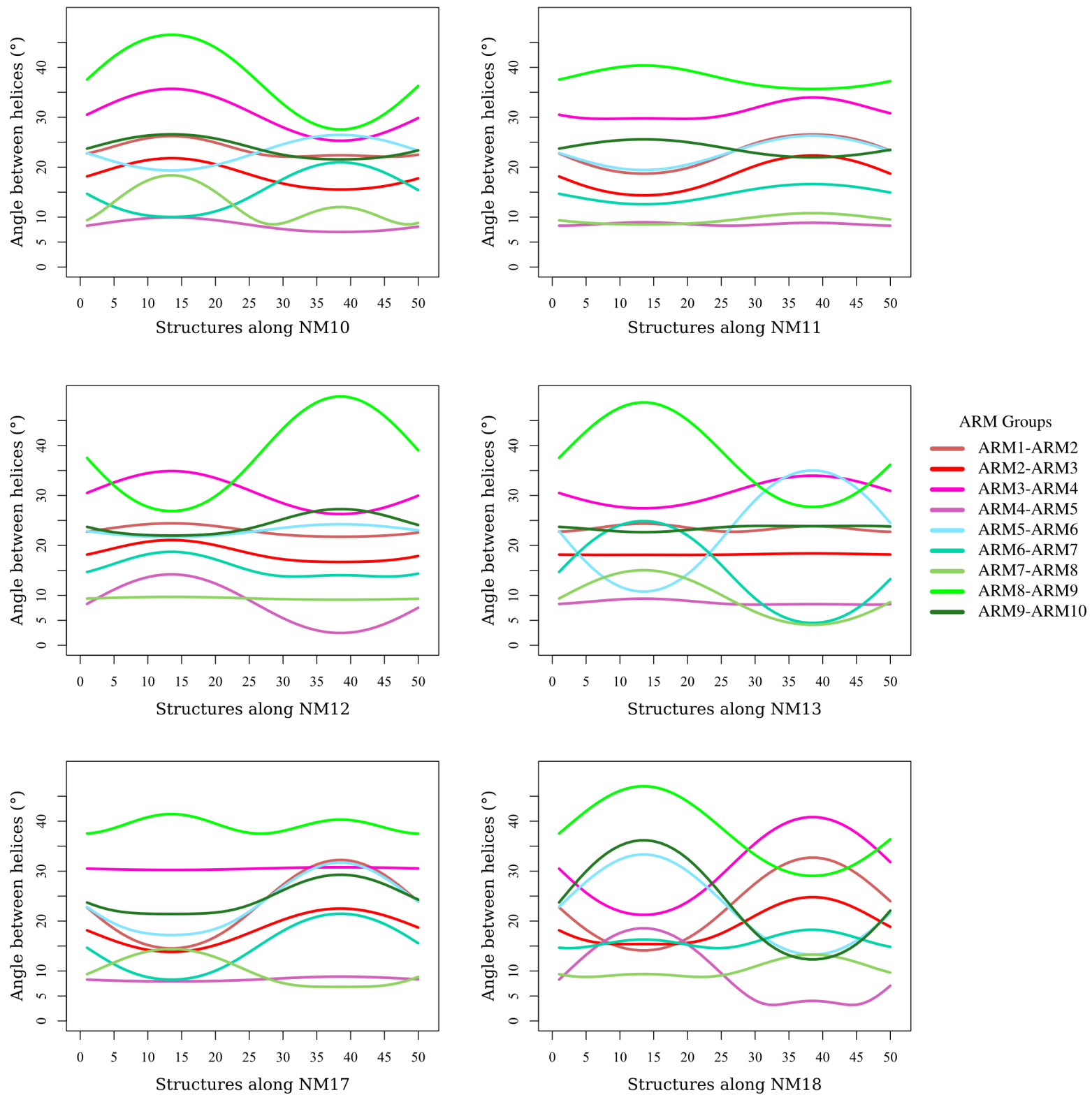

Supplement: S13 Fig — The ARM groups considered for each angle calculation are depicted with different color assignments, similar to Fig 5. (PDF) [file pone.0157162.s016.pdf]
