# Supplementary material for: Bending-Twisting Motions and Main Interactions in Nucleoplasmin Nuclear Import
Source: PLoS One. 2016 Jun 3;11(6):e0157162. doi: 10.1371/journal.pone.0157162 (PMC4892583; doi:10.1371/journal.pone.0157162)
Supplement: S1 Table — The reference structures are aligned with the Impα -NplNLS crystallographic structure (PDB ID 3UL1). (PDF) [file pone.0157162.s017.pdf]

S1 Table: Backbone RMSD values (Å) from structural alignment. The reference structures are aligned with Impα-NpINLS crystallographic structure (PDB ID 3UL1).

**Backbone RMSD**	**67,730 ps**	**207,080 ps**	**274,970 ps**
**Impα**	2.12439	1.15923	2.482
**NpINLS**	1.5432	0.759316	1.70461
