# Supplementary material for: Bending-Twisting Motions and Main Interactions in Nucleoplasmin Nuclear Import
Source: PLoS One. 2016 Jun 3;11(6):e0157162. doi: 10.1371/journal.pone.0157162 (PMC4892583; doi:10.1371/journal.pone.0157162)
Supplement: S2 Table — The occupancies of salt bridges between NplNLS and Impα in standard MD and NM-displacement. Interactions that were above 50% of occupancy are highlighted in gray. (PDF) [file pone.0157162.s018.pdf]

S2 Table: The occupancies of salt bridges between NplNLS and Impα in standard MD and NM-displacement. Interactions that were above 50% of occupancy are highlighted in gray.

Salt Bridges – Standard MD		
NLS	Impα	Occupancies (%)
R156	E396	99.97
D172	R101	64.98
D172	K102	44.85
E153	R117	0.47
K155	D325	2.16
K161	D280	51.68
K162	E354	7.24
K167	D192	96.82
K168	D270	4.00
K168	E266	1.50
K169	E107	4.75
K170	E180	20.04

Salt Bridges – NM displacement		
NLS	Impα	Occupancies (%)
R156	D433	0.06
R156	E396	99.94
D160	R117	0.38
D160	H203	0.06
D172	R101	33.29
D172	R106	0.04
D172	R117	0.02
D172	R227	0.84
D172	H177	0.02
D172	K102	26.99
D172	K108	0.36
E153	R117	1.63
E153	K108	0.06
K155	D325	93.03
K161	D280	99.77
K161	E354	0.02
K162	E354	43.61
K167	D192	98.81
K167	E107	0.04
K168	D270	74.51
K168	E266	100.00
K169	D113	0.08
K169	D192	0.04
K169	E107	33.08
K169	E266	0.88
K169	E306	0.04
K170	E180	80.12
K170	E266	0.33
