# Supplementary material for: Bending-Twisting Motions and Main Interactions in Nucleoplasmin Nuclear Import
Source: PLoS One. 2016 Jun 3;11(6):e0157162. doi: 10.1371/journal.pone.0157162 (PMC4892583; doi:10.1371/journal.pone.0157162)
Supplement: S3 Table — The occupancies of hydrogen bonds between NplNLS (blue) and Impα (green) in standard MD and NM-displacement. Interactions that were above 50% of occupancy are highlighted in gray. (PDF) [file pone.0157162.s019.pdf]

S3 Table: The occupancies of hydrogen bonds between NplNLS (blue) and Impα (green) in standard MD and NM-displacement. Interactions that were above 50% of occupancy are highlighted in gray.

Hydrogen Bonds – Standard MD		
Donor	Acceptor	Occupancies (%)
R156-Side	E396-Side	99.94%
W357-Side	R156-Main	99.61%
G164-Main	Y277-Side	98.62%
R315-Side	T160-Main	98.45%
R156-Main	N361-Side	98.19%
K167-Side	D192-Side	96.58%
K168-Main	N188-Side	96.12%
K155-Side	V321-Main	93.05%
W184-Side	K168-Main	92.35%
W231-Side	Q165-Main	92.13%
N188-Side	K168-Main	85.79%
K167-Side	G150-Main	84.58%
N361-Side	R156-Main	84.33%
R238-Side	G164-Main	83.91%
K167-Side	A148-Main	77.50%
K155-Side	G323-Main	73.12%
R101-Side	D172-Side	65.59%
V154-Main	N403-Side	64.45%
K167-Main	N188-Side	64.25%
N146-Side	K170-Main	64.21%
K170-Main	N146-Side	62.96%
K155-Side	N361-Main	56.44%
K167-Side	T155-Side	52.82%
N403-Side	V154-Main	52.75%
K161-Side	D280-Side	51.89%
R156-Side	S360-Side	50.64%
W142-Side	K170-Main	47.02%
K170-Side	Q181-Side	46.69%
K102-Side	D172-Side	45.34%
K155-Main	N361-Side	44.13%
K167-Side	N188-Main	43.02%
A153-Main	N403-Side	38.06%
N235-Side	A166-Main	37.63%
W231-Side	A166-Main	32.86%
R315-Side	T160-Side	30.54%
K170-Side	W142-Side	29.99%
G191-Main	A166-Main	26.68%
S149-Side	K168-Main	25.98%
A163-Side	Y277-Side	25.12%
K155-Side	T322-Main	24.25%
K168-Side	N228-Side	23.82%
K155-Side	T328-Side	23.68%
W184-Side	K169-Main	22.01%
K167-Side	S149-Main	21.88%

Hydrogen Bonds – NM displacement		
Donor	Acceptor	Occupancies (%)
K168-Side	E266-Side	99.98%
R156-Side	E396-Side	99.92%
K161-Side	D280-Side	99.81%
K167-Side	D192-Side	98.81%
K167-Side	G150-Main	98.37%
R238-Side	Q165-Main	97.47%
K167-Side	A148-Main	96.53%
G164-Main	Y277-Side	96.46%
W357-Side	R156-Main	94.67%
R238-Side	G164-Main	93.39%
K155-Side	D325-Side	92.99%
R156-Side	S360-Side	92.18%
K155-Side	V321-Main	89.64%
R315-Side	T160-Main	88.91%
K155-Side	T328-Side	86.59%
W184-Side	K168-Main	85.40%
N361-Side	R156-Main	84.44%
W231-Side	Q165-Main	83.74%
K170-Side	E180-Side	80.15%
K162-Side	N350-Side	75.31%
K168-Side	D270-Side	74.27%
R156-Main	N361-Side	72.24%
K155-Side	G323-Main	72.07%
R101-Side	D172-Side	66.44%
V154-Main	N403-Side	64.96%
N188-Side	K168-Main	63.24%
K353-Side	T160-Side	58.60%
K102-Side	D172-Side	54.10%
G151-Main	N446-Side	52.38%
N403-Side	V154-Main	51.05%
W231-Side	A166-Main	46.61%
K162-Side	E354-Side	44.25%
N403-Side	R156-Side	42.22%
K170-Side	Q181-Side	42.20%
W142-Side	K170-Main	41.57%
A158-Main	W357-Side	41.53%
R156-Side	N361-Side	39.87%
A153-Main	N403-Side	39.35%
Q165-Side	D270-Side	37.95%
G151-Main	S406-Main	37.89%
K168-Main	N188-Side	37.41%
K167-Side	N188-Side	37.22%
G151-Main	D442-Side	36.80%
N146-Side	K170-Main	36.49%

K170-Side	E180-Side	19.43%
K169-Side	S149-Side	18.51%
K167-Side	N188-Side	17.89%
G151-Main	D442-Side	17.75%
K170-Side	W184-Side	16.62%
A166-Side	G191-Main	15.84%
K168-Side	W231-Side	15.12%
W273-Side	K162-Main	14.36%
K162-Side	N350-Side	13.81%
L171-Side	S105-Main	13.52%

S406-Side	S152-Main	34.35%
G151-Main	G407-Main	34.23%
T160-Side	E354-Side	33.56%
W184-Side	K169-Main	33.35%
K169-Side	E107-Side	33.33%
K168-Main	W231-Side	32.18%
K108-Side	D172-Side	31.97%
Q165-Side	E266-Side	30.90%
K169-Side	N146-Side	29.69%
S149-Side	K168-Main	29.64%
