# Supplementary material for: Bending-Twisting Motions and Main Interactions in Nucleoplasmin Nuclear Import
Source: PLoS One. 2016 Jun 3;11(6):e0157162. doi: 10.1371/journal.pone.0157162 (PMC4892583; doi:10.1371/journal.pone.0157162)
Supplement: S4 Table — The occupancies of hydrophobic contacts between NplNLS and Impα in standard MD and NM-displacement. Interactions that were above 50% of occupancy are highlighted in gray. (PDF) [file pone.0157162.s020.pdf]

S4 Table: The occupancies of hydrophobic contacts between NpINLS and Impα in standard MD and NM-displacement. Interactions that were above 50% of occupancy are highlighted in gray.

Hydrophobic Contacts – Standard MD			Hydrophobic Contacts – NM displacement		
NLS	Impα	Occupancies (%)	NLS	Impα	Occupancies (%)
S152	S406	2.41	S152	S406	0.00
A153	A364	50.61	A153	A364	41.89
A153	N403	33.91	A153	N403	47.00
K155	T322	7.59	K155	T322	10.84
K155	A364	81.50	K155	A364	94.64
R156	W357	1.26	R156	W357	4.02
R156	W399	99.18	R156	W399	98.83
P157	W357	0.00	P157	W357	0.04
A158	R315	83.98	A158	R315	61.75
A158	W357	79.05	A158	W357	95.65
K162	W273	67.96	K162	W273	83.45
K162	T311	0.00	K162	T311	9.50
K162	R315	54.59	K162	R315	14.73
K162	E354	1.43	K162	E354	29.15
A163	W273	0.00	A163	W273	0.31
A163	Y277	70.85	A163	Y277	73.66
A166	N235	0.03	A166	N235	0.04
K167	S149	0.37	K167	S149	17.20
K167	D192	63.47	K167	D192	27.62
K167	W231	0.00	K167	W231	0.69
K168	W184	43.16	K168	W184	17.81
K168	W231	93.57	K168	W231	99.87
K169	N146	35.65	K169	N146	27.18
K170	W142	97.21	K170	W142	86.40
K170	Q181	42.04	K170	Q181	32.45
K170	W184	94.56	K170	W184	96.34
L171	S105	20.54	L171	S105	34.61
L171	R106	0.00	L171	R106	0.04
L171	E107	12.21	L171	E107	30.13
D172	W142	11.33	D172	W142	46.60
