# Supplementary material for: Bending-Twisting Motions and Main Interactions in Nucleoplasmin Nuclear Import
Source: PLoS One. 2016 Jun 3;11(6):e0157162. doi: 10.1371/journal.pone.0157162 (PMC4892583; doi:10.1371/journal.pone.0157162)
Supplement: S5 Table — PDB IDs: 1pjm, 1pjn, 3tpm, 3ukw, 3ukx, 3uky, 3ukz, 3ul1 and 3uvu. (PDF) [file pone.0157162.s021.pdf]

S5 Table. Radius of curvature and average angles for the crystallographic structures.

X-ray PDB ID	Radius of curvature (Å)	Average angle (°)
1pjm	30.993009873	19.6785642989
1pjn	31.2163002693	19.6892134803
3tpm	31.2949958837	19.6517732094
3ukw	31.4596203567	19.2809651455
3ukx	31.1706523738	19.7180289396
3uky	31.2620465432	19.6859686016
3ukz	31.5329175935	19.5667871962
3ul1	30.959989878	19.8467057138
3uvu	31.33650329	19.573789442
